# Indene and indole-based compounds as potential antimicrobial agents: synthesis, activity, docking studies and ADME analysis

**DOI:** 10.1039/d5ra08239k

**Published:** 2026-02-23

**Authors:** Vilma Lovrinčević, Monika Šabić Runjavec, Nikica Baričević, Ines Despotović, Jerome Le-Cunff, Dragana Vuk, Marija Vuković Domanovac

**Affiliations:** a Department of Organic Chemistry, University of Zagreb Faculty of Chemical Engineering and Technology Trg Marka Marulića 19 HR-10000 Zagreb Croatia dvuk@fkit.unizg.hr; b Department of Industrial Ecology, University of Zagreb Faculty of Chemical Engineering and Technology Trg Marka Marulića 19 HR-10000 Zagreb Croatia mvukovic@fkit.unizg.hr; c Division of Physical Chemistry, Ruđer Bošković Institute Bijenička cesta 54 HR-10000 Zagreb Croatia; d Xellia Ltd Slavonska Avenija bb 10000 Zagreb Croatia

## Abstract

The excessive use of antibiotics in recent years has led to an accelerated development of resistance in bacterial pathogens and thus to one of the greatest problems of our time: antibiotic resistance. Therefore, despite the large number of available drugs, the development of new and structurally diverse antibiotics is urgently needed. In this study, various indole and indene derivatives were prepared and characterised and their antibacterial activity against Gram-positive bacteria *Bacillus subtilis* 3020 and Gram-negative bacteria *Pseudomonas aeruginosa* 3011 was investigated. Two fungal strains, *Candida lipolytica* 59 and *Aspergillus niger* 405, were used for antifungal activity. In general, most of the prepared compounds showed potential antifungal activity and antibacterial activity against *Bacillus subtilis* 3020, while all compounds were inactive against *Pseudomonas aeruginosa* 3011. The most promising compounds were pyrrole, pyridine and phenol derivatives, which showed antibacterial and antifungal activity. In addition, molecular docking studies showed that the most promising indole and indene derivatives exhibited significant binding interaction networks and binding affinity with DNA gyrase B (GyrB) and 14α-sterol demethylase (CYP51), consistent with their observed antibacterial and antifungal activities. Finally*, in silico* ADME predictions indicated acceptable physicochemical properties of the newly designed compounds.

## Abstract 2

Various indene derivatives are presented and their antimicrobial activities against different microbial species are evaluated. To elucidate the plausible inhibitory mechanism *in silico* molecular docking study was performed for selected molecules.

## Graphical abstract

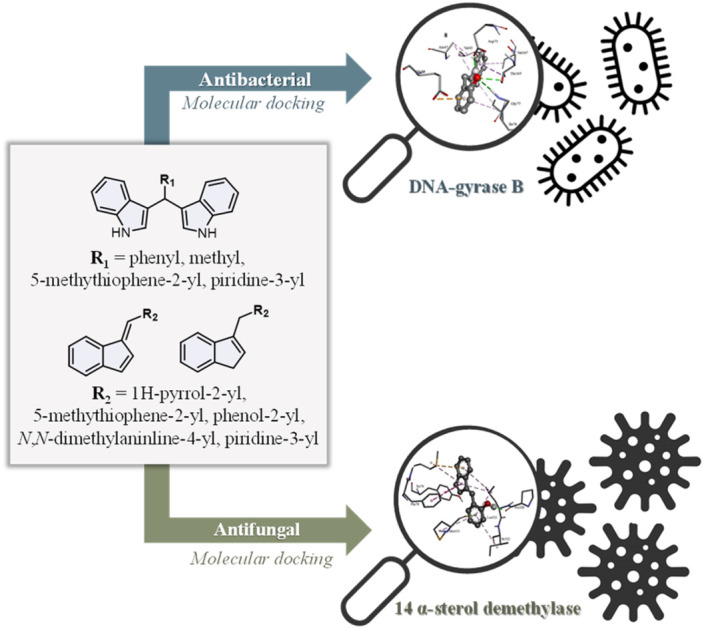

## 1. Introduction

In recent decades, the inappropriate and excessive use of antibiotics has accelerated the development of resistance in bacterial pathogens.^1,2^ The increasing incidence of antibiotic-resistant bacteria has become a major global health problem that requires the search for new and effective antimicrobial agents.^3^ Indanes, indenes and indoles are an important group of organic compounds found in many natural and synthetic products that have shown promise in the fight against bacterial infections due to their diverse chemical structure and unique pharmacological properties.^4,5^ These benzocyclic compounds, which consist of a benzene ring linked to a five-membered ring, could play a key role in the development of new drugs and biologically active precursors. The five-membered ring can be saturated or unsaturated, heterocyclic or non-heterocyclic, with the functional groups determining the class of the compound and its biological activity. Biological effects such as antibacterial, antiviral, antitumor, antioxidant, anti-inflammatory and antidepressant effects are only part of the broad spectrum of these compounds.^6–8^ The ability to modify their structure enables the synthesis of a wide range of derivatives with different functional groups, allowing precise tuning of their chemical and biological activities.^8^ In addition, these compounds have been shown to selectively target bacterial cells, which could reduce the risk of adverse effects on human cells. This makes them promising candidates in the fight against bacterial infections,^9^ especially those caused by antibiotic-resistant compounds.

**Fig. 1:**
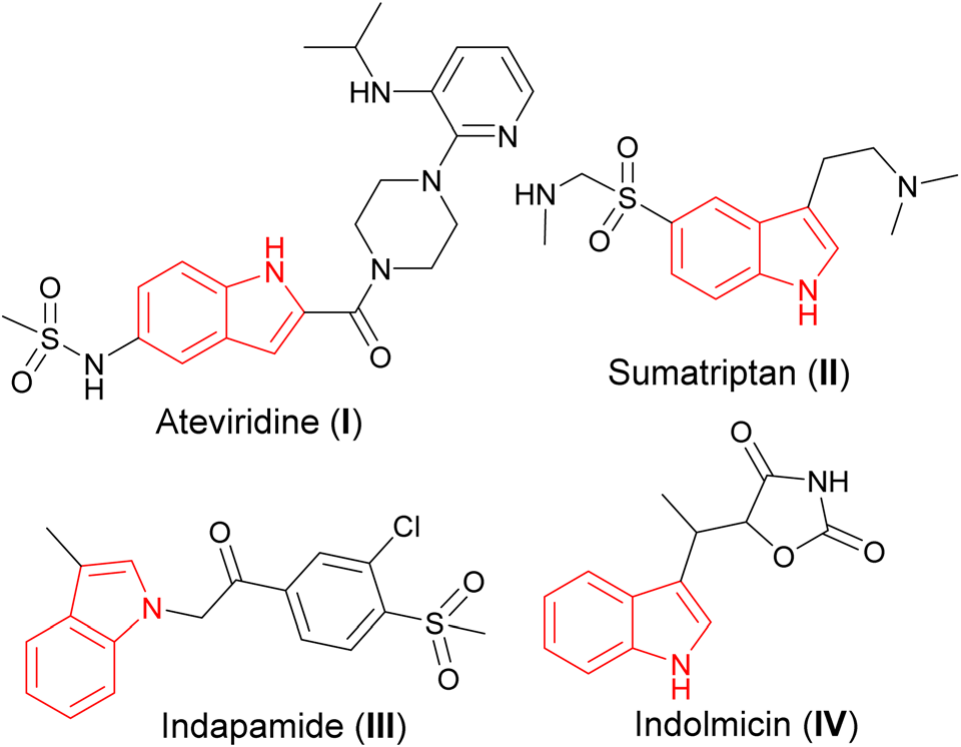
Bioactive indole derivatives.

Indoles with their intriguing structure are known to occupy a special place in synthetic and medicinal chemistry (Fig. 1) and represent an inexhaustible source of new structural modifications with new properties for medical and industrial applications.^10–13^

**Fig. 2:**
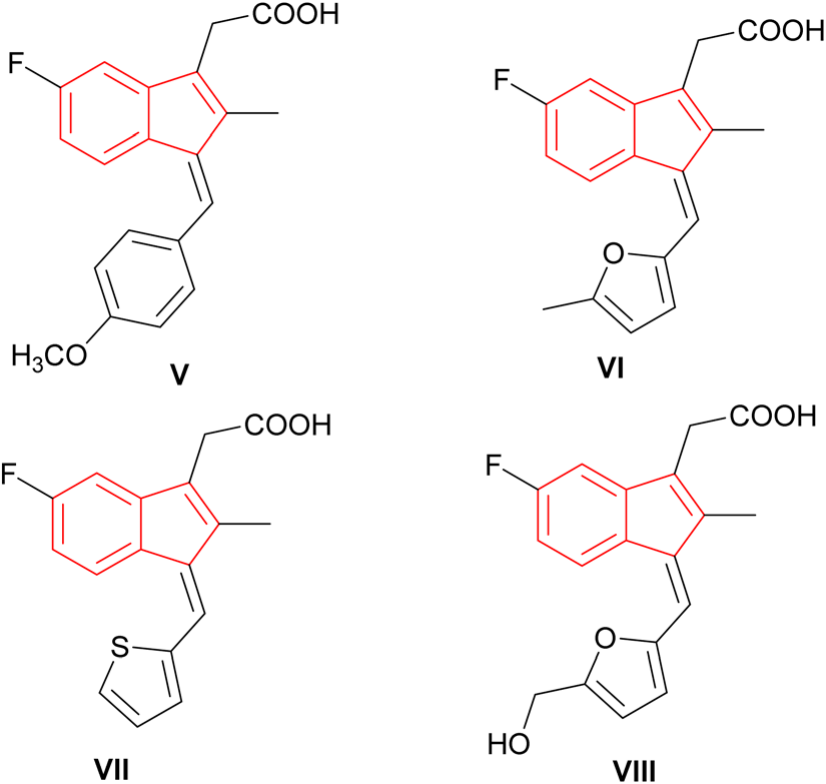
Bioactive indene-based compounds.

Furthermore, indenes due to a number of natural and synthetic compounds, which display pharmaceutical activity, constitute an important class of molecules (Fig. 2). The nature and positioning of the substituents on the indene framework and its appendages determine the structural rigidity or flexibility to achieve an optimised structure with a desired activity profile considered for the regulation of a biological target.^14,15^

In this work, the synthesis of substituted indene and indole derivatives is presented and its antimicrobial activities against different microbial species, *Bacillus subtilis* 3020, *Pseudomonas aeruginosa* 3011, *Candida lipolytica* 59 and *Aspergillus niger* 405, are evaluated. In an effort to elucidate the plausible inhibitory mechanism by which these compounds could be used as antibacterial and antifungal drug candidates, the *in silico* molecular docking study^16^ was performed for selected molecules against the active site of DNA gyrase B (GyrB) and 14α-sterol demethylase (CYP51). The structures of the complexes formed between the potential inhibitor and the target enzyme were predicted, allowing the identification of the main interactions responsible for the inhibitory activity and the estimation of the binding affinity of the active molecule to the target enzyme. Finally, to gain insight into the pharmacokinetics of the newly synthesized compounds, the ADME (absorption, distribution, metabolism, and excretion) properties were calculated. Since heterocyclic compounds themselves could be found as an integral part of biologically active derivatives, the aim was to introduce different heterocyclic nuclei into the existing indene system. Although the considered compounds were prepared according to known procedures, the antimicrobial activity was investigated for the first time, whereby the obtained results could serve as an excellent indicator for future mechanistic studies.

## 2. Results and discussion

### 2.1. Chemistry

#### 2.1.1 Synthesis of indenes 1–9

**Scheme 1:**
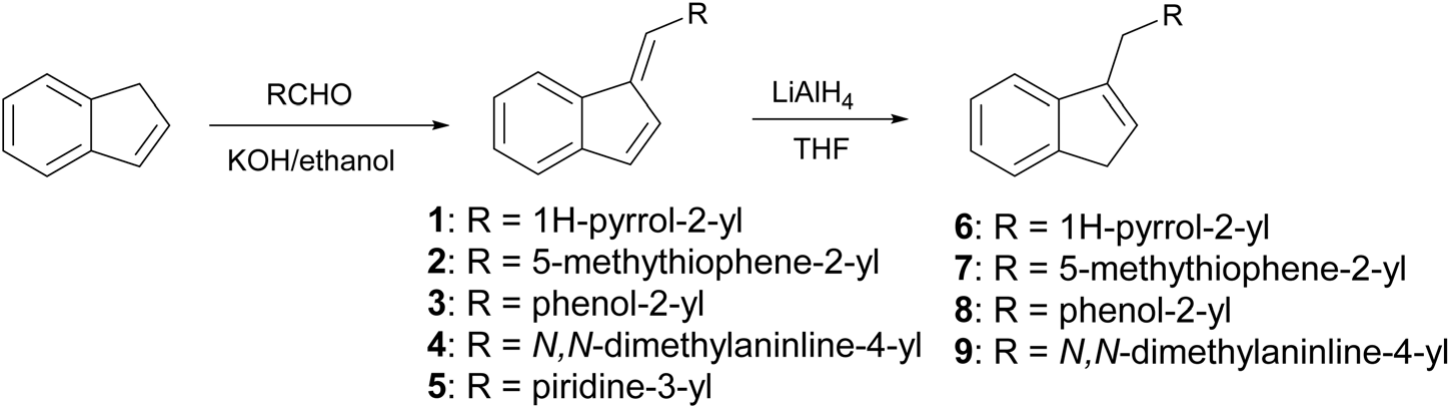
Synthesis of indene derivatives 1–9.

Indene derivatives 1–9 were prepared according to a known procedure^17,18^ staring from indene and corresponding aldehyde. Products 1–5 were obtained by heating indene and aldehyde in KOH/ethanol solution for 3–28 h, followed by reduction with LiAlH_4_ to indenes 6–9 (Scheme 1), respectively. Furthermore, during the synthesis of compounds 3–5, the presence of an unidentified compound was observed in trace. We assume that it could be a stereoisomer of those compounds, which could not be isolated nor purified due to small amounts.

#### 2.1.2 Synthesis of indoles 10–13

**Scheme 2:**
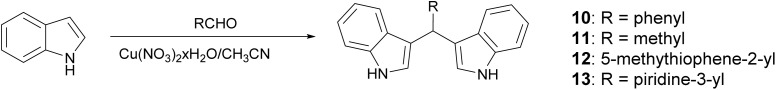
Synthesis of indole derivatives 10–13.

Selected indoles were prepared by condensation of indole and corresponding aldehyde in the presence of Cu(NO_3_)_2_·3H_2_O as catalyst.^19^ This method was presented as a practical procedure for the synthesis of bis(indolyl)-methanes with several advantages as the readily available and inexpensive catalyst, mild reaction conditions, moderate to excellent yields, selectivity, experimental simplicity and study of a wide range of structurally divergent aldehydes. Following the above, products 10–13 were obtained from the mixture of aldehyde and indole at room temperature in the presence of a catalyst (Scheme 2), in moderate yield (12–57%). The lowest yield was observed in the case of the pyridine derivative (13), probably due to the instability of the pyridine nucleus.

### 2.2. Antimicrobial activity

Antimicrobial research is experiencing incredible growth, driven by the urgent need to combat antimicrobial resistance and its impact on global health. Antimicrobial assays are important tools to test the inhibitory effect of numerous compounds against microorganisms. Knowledge of the inhibitory activity of antimicrobial compounds is critical prior to their use.^20,21^ By measuring the resulting zone of inhibition, which represents the area in which microbial growth is prevented or inhibited by the compound, the relative efficacy of the test compound against the specific microorganism tested can be evaluated.^22^

**Fig. 3:**
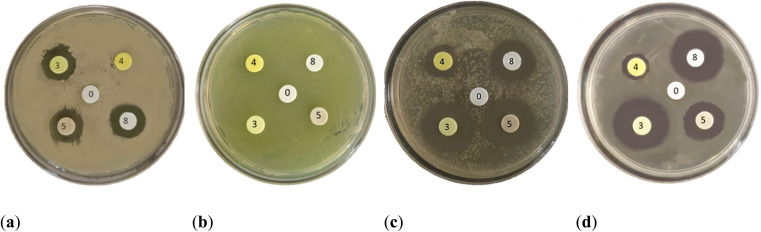
Disk diffusion method of synthesized compounds 3, 4, 5, 8 and 0 as DMSO (control) using test microorganisms: (a) *Bacillus subtilis* 3020; (b) *Pseudomonas aeruginosa* 3011; (c) *Candida lipolytica* 59 and (d) *Aspergillus niger* 405.

The synthesized compounds 1–13 were tested *in vitro* for their antibacterial activities against Gram-positive bacteria *Bacillus subtilis* 3020 and Gram-negative bacteria *Pseudomonas aeruginosa* 3011. Two fungal strains *Candida lipolytica* 59 and *Aspergillus niger* 405 were used to test the antifungal activity. The results of antimicrobial activity for the tested microorganisms are shown in Table 1 and Fig. 3, where the zones around the discs with the tested compounds indicate the antimicrobial activity.

**Table 1:** Antimicrobial activity of the synthesised compounds using the disc diffusion method^a^

Compound	Inhibition zone diameter (mm)			
	*Bacillus subtilis* 3020	*Pseudomonas aeruginosa* 3011	*Candida lipolytica* 59	*Aspergillus niger* 405
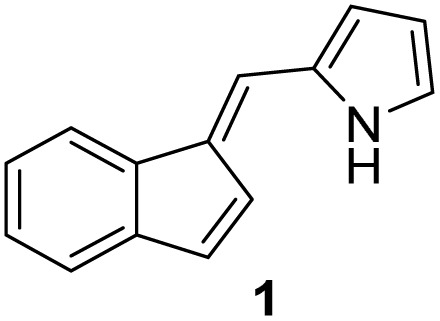	(–)	(–)	20 ± 0.6	26 ± 1.7
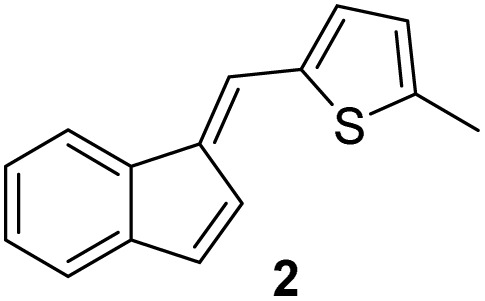	(–)	(–)	(–)	(–)
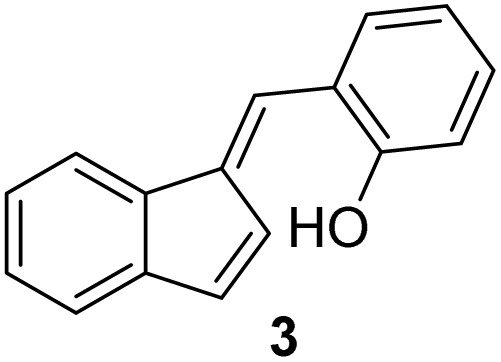	19 ± 1.0	(–)	29 ± 0.6	34 ± 1.5
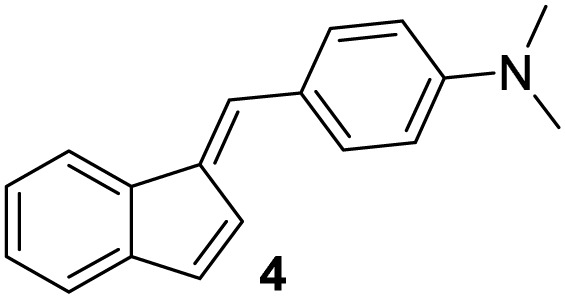	(–)	(–)	17 ± 1.0	13 ± 1.0
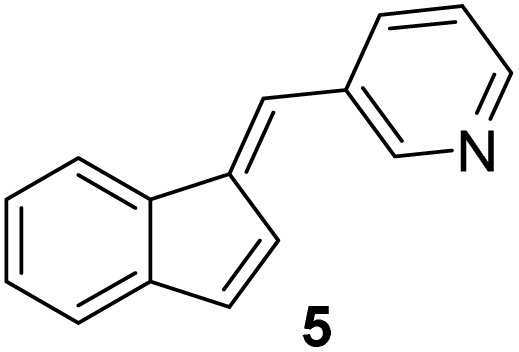	19 ± 0.6	(–)	29 ± 1.2	24 ± 0.6
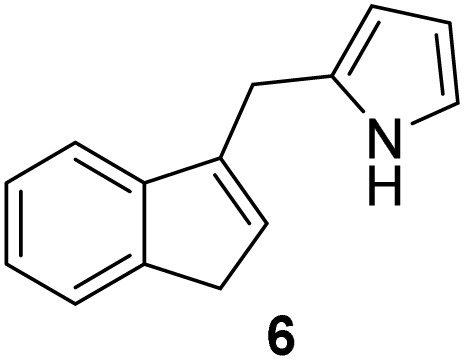	16 ± 1.5	(–)	26 ± 1.5	33 ± 2.1
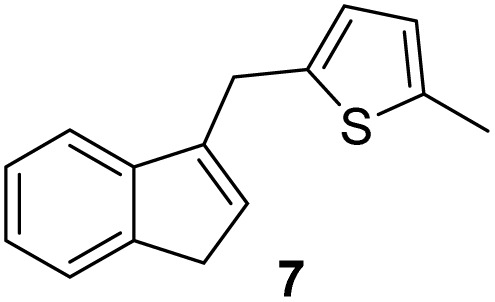	(–)	(–)	15 ± 0.9	19 ± 1.0
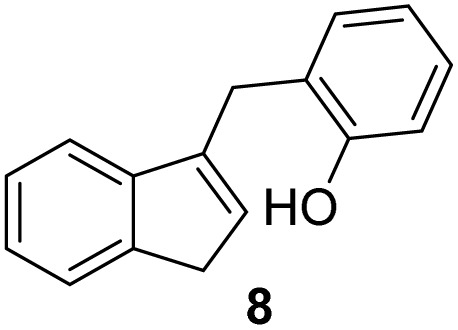	19 ± 0.8	(–)	25 ± 1.2	24 ± 0.6
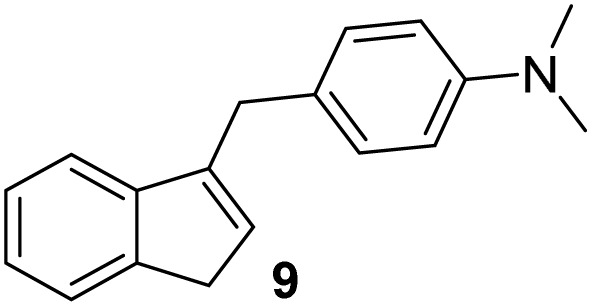	(–)	(–)	22 ± 1.2	27 ± 1.0
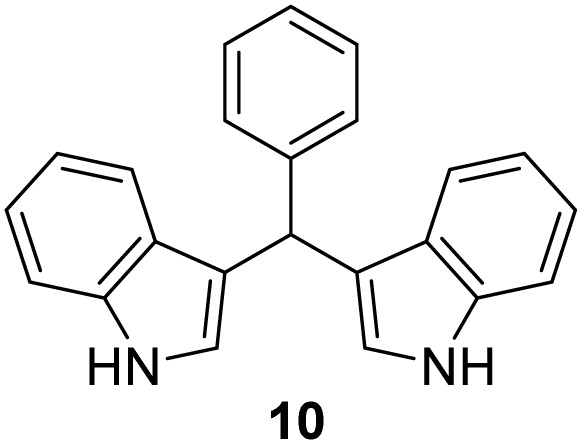	15 ± 0.6	(–)	15 ± 0.5	14 ± 0.6
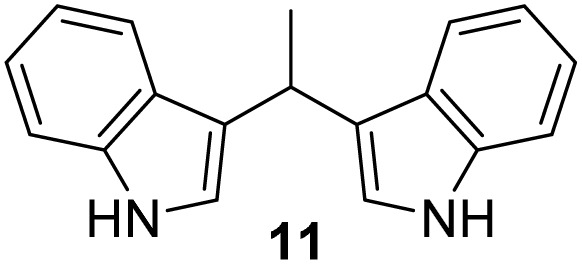	17 ± 0.6	(–)	21 ± 0.5	14 ± 0.9
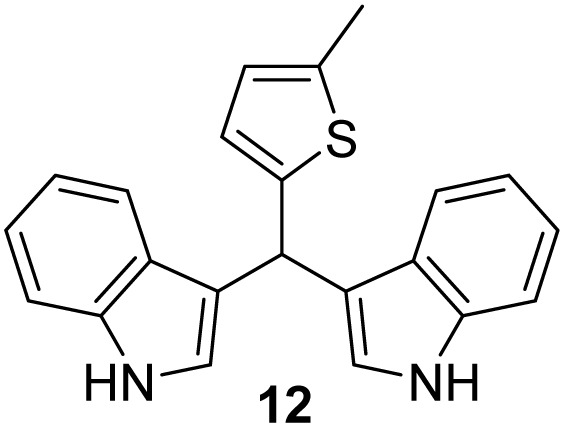	14 ± 0.3	(–)	16 ± 0.6	(–)
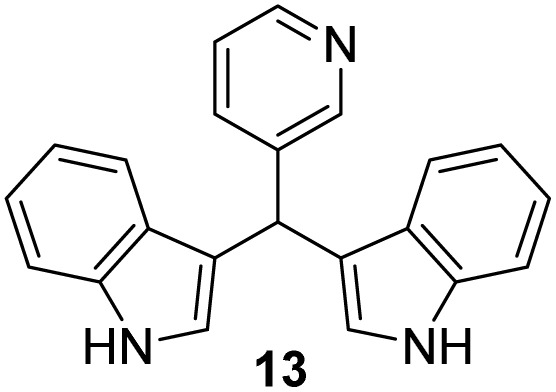	18 ± 1.0	(–)	(–)	(–)
DMSO (control)	(–)	(–)	(–)	(–)
Standard 1	17 ± 1.5	(–)	(–)	(–)
Standard 2	19 ± 0.5	(–)	(–)	(–)
Standard 3	(–)	(–)	25 ± 1.5	20 ± 1.0

a Values are given as mean ± standard error, (−) no activity.

In general, most of the compounds tested showed better activity against Gram-positive than against Gram-negative bacterial species. All tested compounds were found to be inactive against *Pseudomonas aeruginosa* 3011. *P. aeruginosa* is one of the most abundant bacteria in nature and is characterized by its metabolic and physiological versatility, which makes it highly adaptable. *P. aeruginosa* often exhibits extensive intrinsic resistance to a wide range of antimicrobial agents, including tetracyclines and β-lactams.^23^ The development of antimicrobial resistance in *P. aeruginosa* is increasing worldwide, due to the overuse of antibiotics, and exhibits multifactorial mechanisms of response and resistance to antimicrobials*.* Due to its thin peptidoglycan wall, but also its outer membrane, it is difficult for many antibiotics to pass through. *P. aeruginosa* has shown resistance to wide range of antibiotics such as ciprofloxacin, levofloxacin, ceftazidime, imipenem, piperacillin and tazobactam, tobramycin, gentamicin and meropenem.^24^,^25^ The inactivity of the compound against *P. aeruginosa* can be explained by the combination of extremely limited permeability of the outer membrane and strong efflux mechanisms, which together significantly reduce the intracellular accumulation of antimicrobial molecules.^26^ Additionally, more hydrophobic compounds pass through porin channels less efficiently, further limiting entry and contributing to the observed inactivity.^27^

The highest activities towards *Bacillus subtilis* 3020 were observed for indene derivatives 3, 5 and 8, followed by indoles 11 and 13. Compounds 1, 2, 4, 7 and 9 were inactive against *Bacillus subtilis* 3020. *B. subtilis* forms spores and occurs in many natural habitats. It has a thick peptidoglycan wall that is sensitive to many antibiotics. The antibiotic susceptibility tests performed showed that *B. subtilis* 3020 is sensitive to amoxicillin and erythromycin (Table 1), designated as standard 1 and 2. In addition, *B. subtilis* KATMIRA1933 is tolerant to bacitracin and streptomycin and sensitive to penicillin, ampicillin and chloramphenicol.^28^ Studies showed that bacitracin, clindamycin and streptomycin form the smallest inhibition zones against *B. subtilis* SM10.1. After evaluation, *B. subtilis* SM10.1 proved to be resistant to bacitracin, clindamycin and streptomycin, but sensitive to antibiotics such as amoxicillin, chloramphenicol, erythromycin, tetracycline, penicillin and others.^13,29,30^

Antifungal activity was tested with representative microbial models of yeast (*Candida*) and mold (*Aspergillus*) due to their widespread in different environments. By testing different types of fungi, the efficacy of antimicrobials can be evaluated based on the different levels of resistance. According to various studies, *Candida* species have shown sensitivity to amphotericin B and ketoconazole, while resistance to clotrimazole, itraconazole, fluconazole and nystatin has been observed.^31,32^ The genus *Aspergillus* is the causative agent of a large number of diseases in humans. Therefore, testing the susceptibility of *Aspergillus* to antifungals is an important source of information to avoid therapeutic failures. Studies have shown that *A. niger* is sensitive to enilconazole, terbinafine, voriconazole, tioconazole and ketoconazole, less sensitive to clotrimazole, miconazole and nystatin and resistant to amphotericin B, itraconazole, pimaricin, fluconazole and 5-fluorocytosine.^33,34^ Indenes 3, 5 (Fig. 3.) and 6 (Table 1.) showed antifungal activity against both species, *Candida lipolytica* 59 and *Aspergillus niger* 405, and could be the best candidates for further modifications, considering that they also showed antibacterial activity against *Bacillus subtilis* 3020. Both yeast and mold showed antifungal activity against standard 3 ketoconazole. In general, it can be concluded that the presence of indene and indole cores significantly increases antifungal activity, relative to antibacterial activity.

The minimum inhibitory concentration (MIC) was determined using the broth macrodilution method.^35,36^ MIC determination was carried out for selected derivatives 3, 5, 8, 9, 10, and 11 against *Bacillus subtilis* 3020, *Candida lipolytica* 59, and *Aspergillus niger* 405. *Pseudomonas aeruginosa* 3011 was excluded as it was resistant to the compounds. The minimum inhibitory concentration results for the tested microorganisms are presented in Table 2. The MIC results indicate that some indene derivatives, particularly compounds 3 and 8 with lower MIC values, display strong antimicrobial activity against all three tested microorganisms. Susceptibility, also varies significantly between species, with fungal strains in some cases more sensitive than the bacterial strain *B. subtilis*, suggesting selective mechanisms of action for the compounds.

**Table 2:** The minimum inhibitory concentration (MIC) using the broth macrodilution method^a^

Compound	Minimum inhibitory concentration (MIC) (µg mL^−1^)		
	*Bacillus subtilis* 3020	*Candida lipolytica* 59	*Aspergillus niger* 405
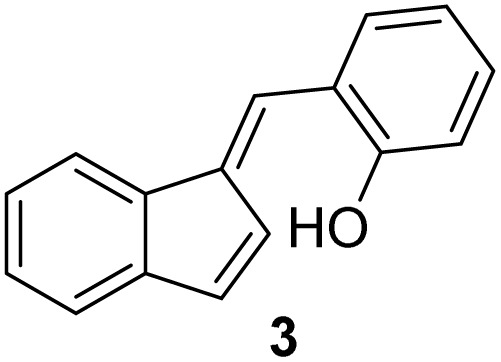	25	2	2
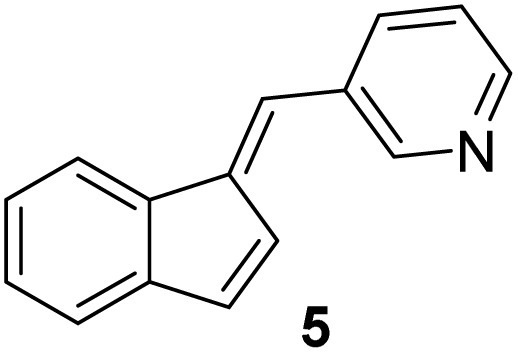	100	25	50
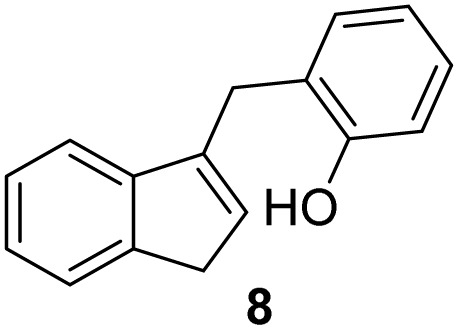	25	4	1
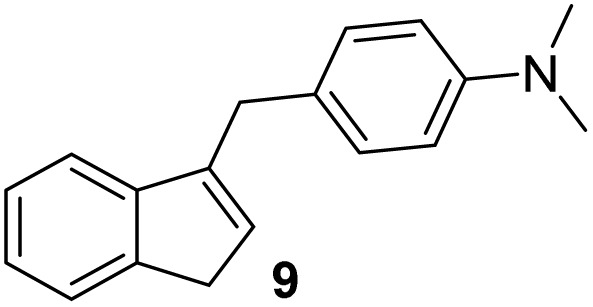	(–)	50	25
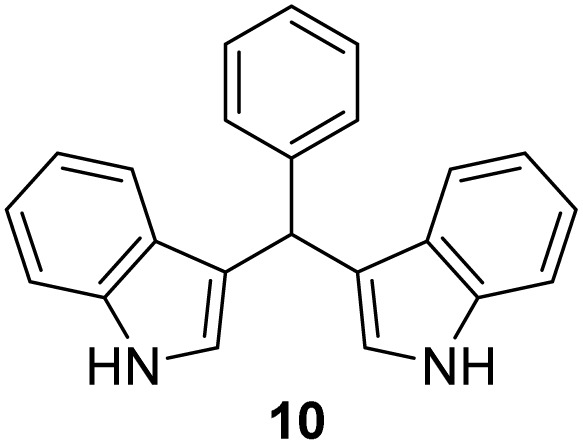	50	13	50
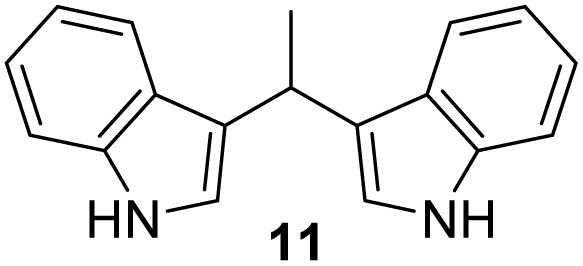	25	7	2

a (−) No activity.

### 2.3. *In silico* molecular docking study

To elucidate the plausible mechanism of antimicrobial activity at the molecular level, *i.e.* to gain insights into the most favorable binding positions of the tested compounds, *in silico* molecular docking simulations were performed. Binding affinities were evaluated and stabilizing interactions were identified in the complexes between the newly synthesized compounds and the active site of respective enzyme through which compounds might exert their antibacterial and/or antifungal activity.

The molecules with significant antimicrobial activity were docked to the enzyme DNA gyrase B (GyrB) (PDB ID: 6F86), a promising antibacterial target that plays a crucial role in ATP hydrolysis and bacterial DNA synthesis^37^ and whose inactivation leads to bacterial death. In addition, the molecules that exhibit significant antifungal activity were docked to 14α-sterol demethylase (CYP51) (PDB ID: 1EA1), a crucial enzyme in eukaryotic sterol biosynthesis,^38^ inhibition of which can disrupt fungal membrane integrity and lead to cell death. Standard molecules, amoxicillin and ketoconazole, were subjected to the docking procedure to the corresponding proteins in order to compare the docking results of the newly synthesized molecules with the corresponding standard.

**Fig. 4:**
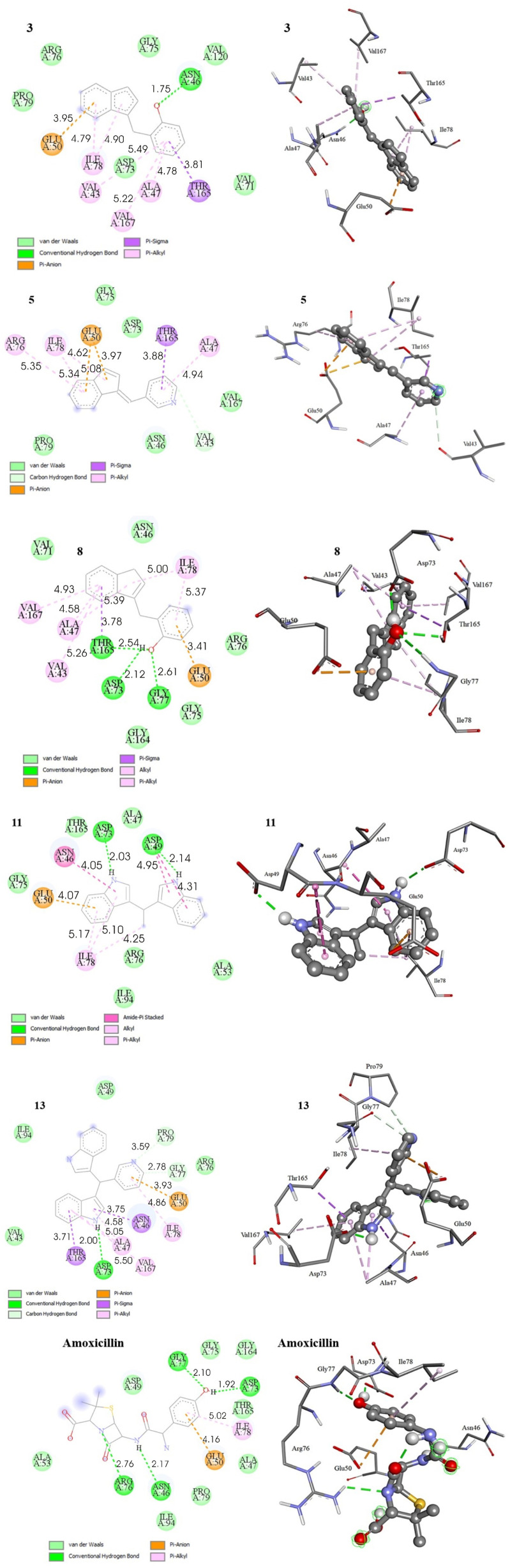
2D and 3D representations of interactions between selected compounds and amino acid residues of DNA gyrase B (PDB ID: 6F86). Hydrogens of the enzyme residues are omitted for clarity.

**Fig. 5:**
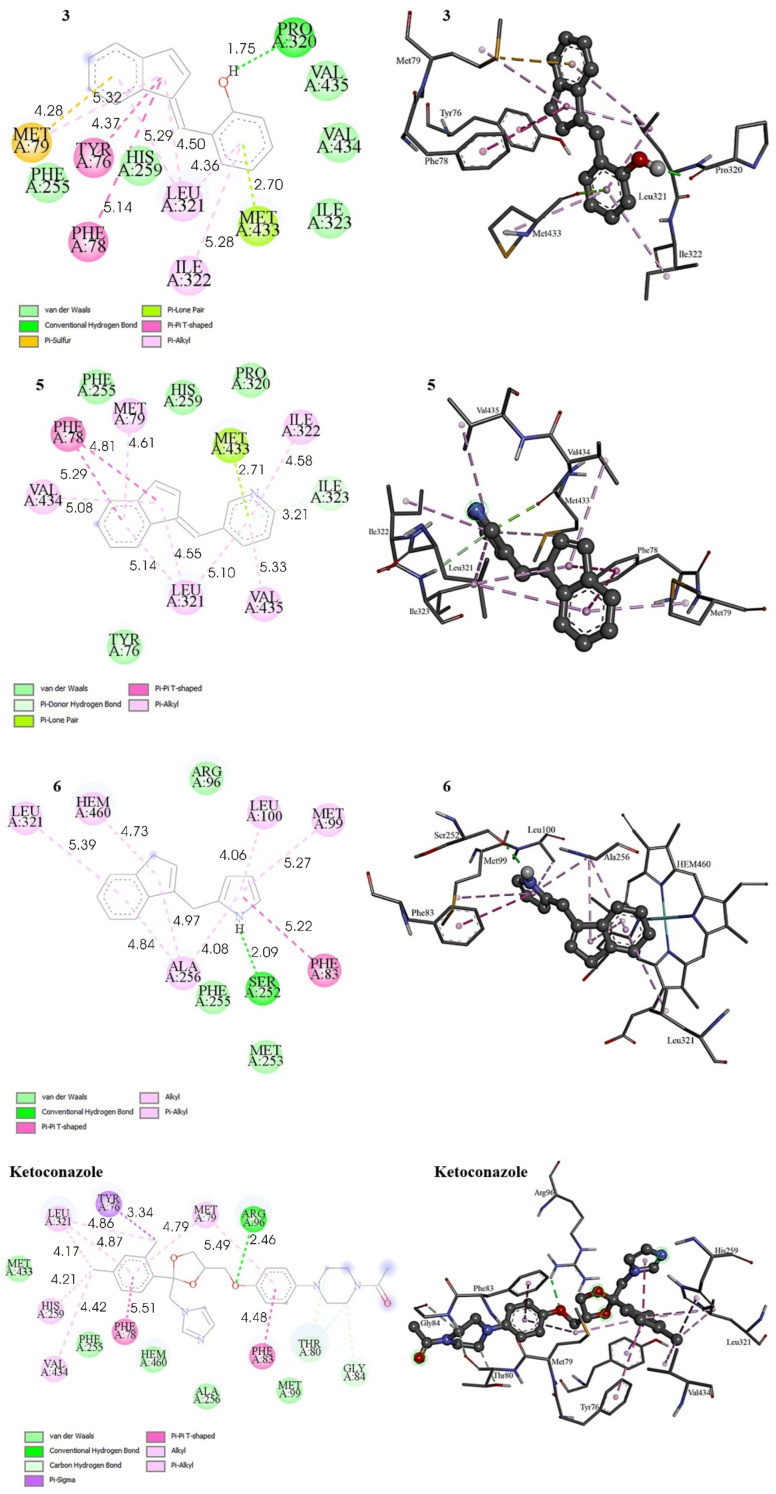
2D and 3D representations of interactions between selected compounds and amino acid residues of 14α-sterol demethylase (CYP51) (PDB ID: 1EA1). Hydrogens of the enzyme residues are omitted for clarity.

The results of molecular docking between the tested ligand and target protein were evaluated through several parameters, including the Gibbs free energy of binding (Δ*G*_bind_), inhibition constant, K*i* and the types of bonds formed between amino acid residues in the active site of the target protein and the selected ligand. The binding energy of the synthesized compounds towards the respective receptor, Δ*G*_bind_, their inhibition constants, *K*_i_, and the residual amino acid interactions with the selected ligands docked to GyrB and CYP51 are listed in Tables 3 and 4. The 2D and 3D representations of interactions between selected compounds or the drug standard, and amino acid residues of the respective target enzyme, obtained by docking, are depicted in Fig. 4 and 5.

**Table 3:** The binding energy (Δ*G*_bind_) in kcal mol^−1^, inhibition constant (*K*_i_), and amino acid residue–ligand interactions for the selected compounds and standard drug amoxicillin targeting DNA gyrase B (GyrB) (PDB ID: 6F86)

Ligand compound	Δ*G*_bind_	*K*_i_ a (µM)	Amino acid residues (interactions)
3	−7.31	4.40	Asn46(Hydrogen bond), Glu50(π–anion), Ile78(π–alkyl), Val43(π–alkyl), Val167(π–alkyl), Thr165(π–sigma), Ala47(π–alkyl), Arg76(van der Waals), Pro79(van der Waals), Gly75(van der Waals), Val120(van der Waals)
5	−6.97	7.81	Glu50(π–anion), Ile78(π–alkyl), Arg76(π–alkyl), Ala47(π–alkyl), Thr165(π–sigma),Val43(Carbon hydrogen bond), Gly75(van der Waals), Asp73(van der Waals), Val167(van der Waals) Asn46(van der Waals), Pro79(van der Waals
8	−6.93	8.30	Thr165(Hydrogen bond, π–sigma), Asp73(Hydrogen bond), Gly77(Hydrogen bond), Glu50(π–anion), Ile78(π–alkyl), Val167(π–alkyl), Ala47(π–alkyl), Val43(π–alkyl), Val71(van der Waals), Asn46(van der Waals), Arg76(van der Waals), Gly75(van der Waals), Gly164(van der Waals)
11	−6.85	9.46	Asp49(Hydrogen bond, amide- π stacking), Asp73(Hydrogen bond), Glu50(π–anion), Ile78(π–alkyl, alkyl), Asn46(amide–π stacking), Gly75(van der Waals), Thr165(van der Waals),Ala47(van der Waals), Arg76(van der Waals), Ile94(van der Waals), Ala53(van der Waals)
13	−7.34	4.17	Asp73(Hydrogen bond), Pro79(Carbon hydrogen bond), Gly77(Carbon hydrogen bond), Glu50(π–anion), Ile78(π–alkyl), Ala47(π–alkyl), Val167(π–alkyl), Thr165(π–sigma), Asn46(π–sigma), Ile94(van der Waals), Arg76(van der Waals), Val43(van der Waals)
Amoxicillin	−5.28	135.77	Asn46(Hydrogen bond), Arg76(Hydrogen bond), Glu50(π–anion), Ile78(π–sigma), Asp73(Hydrogen bond), Gly77(Hydrogen bond), Asp49(van der Waals), Gly75(van der Waals), Gly164(van der Waals), Thr165(van der Waals), Ala47 (van der Waals), Pro79(van der Waals), Ile94(van der Waals), Ala53(van der Waals)

a *K*_i_ = exp(Δ*G*/*RT*), where *R* = 1.985 × 10^−3^ kcal mol^−1^ K^−1^ and *T* = 298.15 K.

**Table 4:** The binding energy (Δ*G*_bind_) in kcal mol^−1^, inhibition constant (*K*_i_), and amino acid residue–ligand interactions for the selected compounds and standard drug ketoconazole targeting 14α-sterol demethylase (CYP51) (PDB ID: 1EA1)

Ligand compound	Δ*G*_bind_	*K*_*i*_ a (µM)	Amino acid residual (interactions)
3	−8.20	0.98	Pro320(Hydrogen bond), Ile322(π–alkyl), Leu321(π–alkyl), Tyr76(π–π T-shaped), Phe78(π–π T-shaped), Met79 (π–sulphur, π–alkyl), Met433(π–lone pair), Phe255(van der Waals), Val435(van der Waals), Val434(van der Waals), His259(van der Waals), Ile323(van der Waals)
5	−8.38	0.72	Met433(π–lone pair), Ile322(π–alkyl), Val435(π-alkyl), Leu321(π-alkyl), Val434(π-alkyl), Met79(π-alkyl), Phe78(π– π T-shaped), Ile323(π-donor hydrogen bond)), Phe255(van der Waals), His259(van der Waals), Pro320(van der Waals), Tyr76 (van der Waals)
6	−7.22	5.13	Ser252(Hydrogen bond), Leu100(π-alkyl), Met99 (π-alkyl), Ala256(π-alkyl), Leu321 (π-alkyl), Hem460(π-alkyl), Phe83(π– π T-shaped), Arg96(van der Waals), Phe255(van der Waals), Met253(van der Waals)
Ketoconazole	−9.45	0.12	Arg96(Hydrogen bond), Thr80(Carbon hydrogen bond), Gly84(Carbon hydrogen bond), Phe83(π–π T-shaped), Phe78(π–π T-shaped), Met79(π–alkyl), Leu321(alkyl, π–alkyl), Val434(alkyl), His259(alkyl), Tyr76(π–sigma), Met433(van der Waals), Phe255(van der Waals), Hem460(van der Waals), Ala256(van der Waals),Met99(van der Waals)

a *K*_i_ = exp(Δ*G*/*RT*), where *R* = 1.985 × 10^−3^ kcal mol^−1^ K^−1^ and *T* = 298.15 K.

Among experimentally evaluated compounds showing antibacterial activity the best results were achieved with compounds 3, 5, 8, followed by 11 and 13. Therefore, those compounds were selected for *in silico* docking analysis against enzyme GyrB (PDB ID: 6F86). As shown by the data given in Table 3 free energies of binding (Δ*G*_bind_) for these compounds exhibit negative values indicating favorable interaction between the protein and the ligand. They span the range from −7.34 kcal mol^−1^ for compound 13 to −6.85 kcal mol^−1^ for compound 11, all of that being higher than the value evaluated for standard drug amoxicillin of −5.28 kcal mol^−1^. In addition, the inhibition constant of the selected compounds (*K*_i_), which is actually a dissociation constant of the docked enzyme–inhibitor complex, was calculated to span a range between 4.17 and 9.46 µM, giving lower values compared to amoxicillin, which had an inhibition constant of 135.77 µM (Table 3). The lower value of *K*_i_ indicates a lower probability of dissociation and thus a higher inhibition. As the results show, all compounds tested were more potent than the standard drug amoxicillin, with indole derivative 13 and indene derivative 3 being the most potent.

As depicted in Fig. 4, compound 3 exhibited one hydrogen bonding interaction with amino acid residue Asn46, several hydrophobic interactions with Ile78, Val43, Ala47, Val167, and Thr165, and π–anion interaction with Glu50. van der Waals interactions are established with Arg76, Pro79, Gly75 and Val120. Inhibitory activity shown by compound 5 were accomplished through carbon hydrogen bond interaction with Val43, several hydrophobic interactions, with Arg76, Ile78, Thr165 and Ala47, and one π–anion interaction with Glu50. In addition, van der Waals interactions are occurred with Gly75, Asp73, Val167, Asn46 and Pro79. For molecule 8 hydrogen bond interactions with Thr165, Asp73 and Gly77, hydrophobic interactions with Val167, Ala47, Val43, Ile78 and Thr165, one π–anion interaction with Glu50 and van der Waals interactions with Val71, Asn46, Arg76, Gly75 and Gly164 were observed. Indole derivative 11 showed hydrogen bond interactions with Asp49 and Asp43, hydrophobic interactions with Ile78, Asn46 and Asp49, π–anion interaction with Glu50 and several van der Waals interactions with Gly75, Thr165, Ala47, Arg76, Ile94 and Ala53. The protein–derivative 13 stabilized interactions are accomplished through hydrogen bonding with Asp73, carbon hydrogen bonding with Gly77 and Pro79, hydrophobic interactions with Ile78, Val167, Ala47, Asn46 and Thr165, π–anion interaction with Glu50, and van der Waals interactions with Ile94, Arg76 and Val43 amino acid residues. Finally, the amoxicillin drug standard interacts with the targeted protein through four hydrogen bonds with Asn46, Arg76, Asp73 and Gly77 amino acid residues. In addition, hydrophobic interaction with Ile78, electrostatic interaction with Glu50 and van der Waals interactions with Gly75, Gly164, Thr165, Ala47, Pro7, Ile94, Ala53 and Asp49 have been observed.

In general, the molecular docking simulations showed that the tested compounds are accommodated by GyrB mainly *via* hydrophobic interactions in which π–alkyl interactions play an important role, inferring that they are crucial for the efficient binding of the compounds to receptor proteins. In addition, hydrogen bonds established *via* the phenolic hydroxyl group (–OH) or indole –NH group of the ligands with Asn46, Gly77, Asp73, Thr165 and Asp49 (Fig. 4), as well as electrostatic π–anion interactions with the Glu50 amino acid, were found to play an important role in binding to the active site of the target enzyme, thereby enhancing the biological activity of the tested compounds. Furthermore, favorable van der Waals interactions were found to additionally contribute to the overall ligand–protein association at a protein binding site.

Among experimentally tested compounds that exhibited antifungal activity, the most promising results were obtained with compounds 3, 5 and 6, therefore, the *in silico* docking analysis of the respected compounds along with the ketoconazole standard drug against enzyme 14α-sterol demethylase (PDB ID: 1EA1) was carried out. The inspection of the free energies of binding (Δ*G*_bind_) for examined compounds showed that they exhibit significant inhibitory potential with Δ*G*_bind_ values of −8.20 kcal mol^−1^, -8.38 kcal mol^−1^ and −7.12 kcal mol^−1^ for compound 3, 5, and 6, respectively (Table 4). They expressed slightly lower binding energy in comparison with that of ketoconazole standard whose Δ*G*_bind_ is −9.45 kcal mol^−1^, obtained by the same docking procedure, suggesting that those ligands exhibit something lower potential as the inhibitors in comparison of the selected standard drug. The inhibition constant of the selected compounds (*K*_i_) have been calculated to span the range between 0.14 and 5.13 µM, being higher, as expected according to lower binding energies, in comparison with that of ketoconazole, which showed an inhibition constant of 0.12 µM (Table 4).

The structure of the 14α-sterol demethylase active site docked with compound 3, shown in Fig. 5, reveals that 3 establishes the hydrogen bond interaction with Pro320. In addition, the benzene moiety binds with Met433 through π–lone pair interaction and with Ile322 and Leu321 through hydrophobic π–alkyl interaction, while indene moiety binds with Leu321 *via* π–alkyl interaction, with Phe78 and Tyr76 *via* π–π T-shaped interactions and with sulfur in Met79 *via* π–sulfur interaction. Met79 is also engaged in one π–alkyl interaction with five-membered ring of the indene core. Several van der Waals interactions are found with Val435, Val434, Ile323, Phe255 and His259. Compound 5 exhibits five π–alkyl interactions with Ile322, Val435, Leu321, Val434 and Met79, one π–lone pair interaction with Met433, and one π–donor hydrogen bond with Ile323. In addition, indene moiety of the compound 5 binds with Phe78 *via* two π–π T-shaped hydrophobic interactions. van der Waals interactions are established with Phe255, His259, Pro320 and Tyr76, as shown in Fig. 5. The binding pose of 6 projects hydrogen bond interaction with Ser252 residue. In addition, pyrrole moiety of the ligand molecule displays π–alkyl interaction networks with Ala256, Leu100 and Met99 and is involved in perpendicular π–π stacking with Phe83. Likewise, indene moiety of 6 forms four π–alkyl interactions with Ala256 and Leu321 and Hem460 (Fig. 5). van der Waals interactions are established with Arg96, Phe255 and Met253. Finally, standard drug ketoconazole displays one hydrogen bond with Arg96. Benzene moieties of the drug bind with Phe83 and Phe78 *via* π–π T-shaped interaction, and with Met79 and Leu321 *via* π–alkyl interactions. Four alkyl–alkyl interactions are observed with Val434, His259 and Leu321, one π–sigma interaction with Tyr76, while the diazinane core of the ligand is engaged in carbon hydrogen bond with Thr80 and Gly84, as shown in Fig. 5. van der Waals interactions are shown with Met433, Phe255, Hem460, Ala256 and Met99.

According to the molecular docking simulations, hydrophobic interactions in the form of π–alkyl and π–π T-shaped interactions mainly contribute to the binding of the tested molecules to the 14α-sterol demethylase, suggesting that these interactions, may play an important role in the observed biological activity of the considered compounds. In addition, the hydrogen bond stabilizing interactions are also significant, including those that occur *via* the phenolic hydroxyl group (–OH) or the pyrrole –NH group of the molecules under investigation. Some residues interact *via* multiple bonds, and it can be surmised that these residues are crucial for the binding of the protein to the drug molecule.

It should be noted here that docking simulations do not provide direct evidence of enzymatic inhibition or target engagement. Instead, these results should be interpreted as hypothesis-generating, indicating potential molecular interactions that may underlie the observed biological activity. Therefore, while the present findings are consistent with the possibility that GyrB and CYP51 may be relevant molecular targets, validation of these computational hypotheses will require *in vitro* biochemical assays, including enzymatic inhibition and direct binding studies, to determine whether the predicted interactions result in functional inhibition.

#### 2.3.1 *In silico* ADME properties

Drug development and design is a lengthy, labor-intensive, and resource-demanding process. One of the greatest challenges is determining ADME properties in humans, which can now be efficiently predicted using *in silico* tools such as SwissADME^37^ for evaluating the pharmacokinetics, drug-likeness, and medicinal chemistry friendliness of molecules. The synthesized compounds 3, 5, 6, 8, 11 and 13 were analyzed using SwissADME for drug-likeness, following Lipinski's Rule of Five, which establishes physicochemical criteria for a high probability of oral drug success. According to this rule, a drug-like compound should have a molecular weight no greater than 500 Da, high lipophilicity (log *P* value less than 5), no more than 5 hydrogen bond donors, no more than 10 hydrogen bond acceptors, and a maximum total polar surface area (TPSA) of 140 Å^2^. Results obtained from SwissADME predictions show that all the screened compounds satisfy Lipinski's Rule of Five, with zero violations (Table 5). The molecular weights of the compounds ranged from 195.26 to 323.39 Da, while the log *P* values ranged from 3.11 to 4.12, showing desired lipophilicity. The numbers of hydrogen acceptors and donors fall within the appropriate ranges. Additionally, the TPSA values of the studied compounds ranged from 12.89 to 44.47 Å^2^, well below the 140 Å^2^ limit.

**Table 5:** Drug-likeness predictions of docked ligands evaluated by SwissADME^a^

Ligand	Mol. wt. (g mol^−1^)	NRB	NHA	NHD	TPSA (Å^2^)	log *P* (*c* Log *P*)	Lipinski's rule of five violation
3	220.27	1	1	1	20.23	3.50	0
5	205.25	1	1	0	12.89	3.21	0
6	195.26	2	0	1	15.79	3.11	0
8	222.28	2	1	1	20.23	3.55	0
11	260.33	2	0	2	31.58	3.95	0
13	323.39	3	1	2	44.47	4.12	0

a Abbreviations: Mol. wt., molecular weight; NRB, number of rotatable bonds; NHA, number of hydrogen acceptors; NHD, number of hydrogen donors; TPSA, total polar surface area; log *P*, logarithm of *n*-octanol-water partition coefficient; *c* Log *P*, calculated logarithm of *n*-octanol-water partition coefficient.

The skin permeation value (log *K*p) of tested compounds was found to be in the range of −4.74 to −5.35 cm s^−1^, which indicates low skin permeability (Table 6). The *in silico* predictions showed that all the compounds exhibited high gastro-intestinal (GI) absorption and blood–brain barrier (BBB) permeation, suggesting high absorption and distribution. Moreover, indole derivatives 11 and 13 were found to be suitable substrates of permeability glycoprotein (P-gp), whereas indene derivatives were not. It was also found that all the compounds inhibit CYP1A2 and CYP2C19, but none of them exhibit inhibitory interactions with CYP2C9. Compound 3 and 5 neither inhibit CYP2D6 nor CYP3A4. Compound 6 inhibits CYP2D6 but not CYP3A4, a profile it shares with compound 8, while compounds 11 and 13 inhibit both CYP2D6 and CYP3A4 (Table 6).

**Table 6:** Pharmacokinetic predictions of docked ligands evaluated by SwissADME^a^

Ligand	Log *K*_p_ (cm s^−1^)	GI absorption	BBB permeant	Inhibitor interactions					
				P-gp	CYP1A2	CYP2C19	CYP2C9	CYP2D6	CYP3A4
3	−4.84	High	Yes	No	Yes	Yes	No	No	No
5	−5.25	High	Yes	No	Yes	Yes	No	No	No
6	−5.35	High	Yes	No	Yes	Yes	No	Yes	No
8	−4.94	High	Yes	No	Yes	Yes	No	Yes	No
11	−4.74	High	Yes	Yes	Yes	Yes	No	Yes	Yes
13	−5.09	High	Yes	Yes	Yes	Yes	No	Yes	Yes

a Abbreviations: GI, gastro-intestinal; BBB, blood–brain barrier; P-gp, P-glycoprotein; CYP, cytochrome-P.

Although the predicted ADME parameters above fall within commonly accepted drug-like ranges, these results should be interpreted with caution, as they provide only preliminary computational estimates of pharmacokinetic feasibility and cannot substitute for experimental ADME or *in vivo* pharmacological studies; therefore, experimental validation is required.

## 3. Materials and methods

### 3.1. General experimental information

The NMR spectra were recorded with a spectrometer from Bruker (Germany) on the AV600 and AV300. The ^1^H NMR spectra were recorded at 300 and 600 MHz and the ^13^C NMR spectra at 75 and 150 MHz. All NMR spectra were measured in CDCl_3_ using tetramethylsilane as reference. The assignment of the signals is based on 2D-CH correlation and 2D-HH-COSY and NOESY experiments. Silica gel 0.063–0.2 mm (Fluka, Switzerland) was used for chromatographic purification. The following chemicals were used: indene (Fluka Chemika, Switzerland), indole (MERCK, Germany), pyrrole-2-carbaldehyde (Fluka Chemika, Switzerland), 5-methylthiophene-2-carbaldehyde (Aldrich, Germany), salicylaldehyde (REACHIM, Russia), *para*-dimethylaminobenzaldehyde (Kemika, Croatia), pyridine-3-carbaldehyde (MERCK, Germany), benzaldehyde (MERCK, Germany), acetaldehyde (MERCK, Germany), tetrahydrofuran (THF) (Thermo Scientific, Germany), lithium aluminium hydride (Aldrich, USA), Cu(NO_3_)_2_·3H_2_O (MERCK, Germany). Amoxicillin, erythromycin and ketoconazole were used as standard 1, 2 and 3 (Sigma Aldrich, USA).

### 3.2. Antimicrobial activity

For antimicrobial testing, the disc diffusion method was used, which was performed in accordance with the Clinical Laboratory Standards Institute (CLSI).^39^ The following microorganisms were used in this work: Gram-positive bacteria *Bacillus subtilis* 3020, Gram-negative bacteria *Pseudomonas aeruginosa* 3011 and fungi *Candida lipolytica* 59 and *Aspergillus niger* 405. All microorganisms are kept in the Microorganism Collection of the University of Zagreb Faculty of Chemical Engineering and Technology, Croatia. The bacterial cultures were grown on nutrient agar at 37 °C and the fungal cultures on malt agar at 28 °C. All media for microbial analysis were purchased from Biolife, Italy. Prior to antimicrobial activity testing, all microbial cultures were freshly grown for 24 h, with the exception of the fungus *Aspergillus niger* 405, which was grown for 72 h.

20 mL of Mueller–Hinton agar (MHA) was poured into a Petri dish. 100 µL of a freshly prepared microbial culture suspension (0.5 McFarland) was applied to the surface of the MHA. 50 µL of each sample (10 mg mL^−1^) was impregnated into 9 mm diameter discs. The Petri dishes were incubated at 37 °C, and the antibacterial activity was determined by measuring the diameter of the zone of inhibition around the discs after 24 and 72 h. The test was repeated three times. The antibacterial activity was expressed as the mean diameter of the zone of inhibition (mm).

### 3.3. General procedure for synthesis of indenes 1–5

Indene and aldehyde (1 eq.) were dissolved in 1% KOH–ethanol and the mixture was heated under reflux for 1.5–72 h. The solution was concentrated under reduced pressure and extracted with dichloromethane and water. The solution was dried over magnesium sulfate and filtered, followed by evaporation of the solvent. The crude reaction mixture chromatographed on a silica gel column using petroleum ether and dichloromethane (3 : 1) as eluents, whereby products 1–5 were obtained and characterized, respectively.

#### 3.3.1 (*E*)-2-((1*H*-Inden-1-ylidene)methyl)-1*H*-pyrrole (1)^17^

The synthesis was carried out according to the general procedure described above from indene (500 mg; 4.3 mmol) and pyrrole-2-carbaldehyde (409 mg, 4.3 mmol) in 1% KOH–ethanol (18 mL) stirred at reflux for 72 h. The product was purified by column chromatography on silica gel using petroleum ether and dichloromethane (3 : 1) as eluents to afford the pure compound 1 as yellow solid.

1: 307 mg (37%); mp 134–135 °C; ^1^H NMR (CDCl_3_, 600 MHz) *δ*/ppm: 8.55 (s, 1H), 7.65–7.61 (m, 1H), 7.35–7.32 (m, 1H), 7.24 (s, 1H), 7.22–7.18 (m, 2H), 7.01–6.98 (m, 3H), 6.68 (s, 1H), 6.36 (dd, *J* = 6.2 Hz; 2.5 Hz, 1H); ^13^C NMR (CDCl_3_; 150 MHz) *δ*/*ppm*: 141.2, 137.8, 133.8, 132.9, 130.3, 126.5, 124.9, 124.8, 122.2, 121.1, 118.6, 118.0, 114.9, 111.4; HRMS (LC-Q/TOF) *m*/*z* [M + H]^+^ calculated for C_14_H_12_N^+^ 194.0964, found 194.0964.

#### 3.3.2 (*E*)-2-((1*H*-Inden-1-ylidene)methyl)-5-methylthiophene (2)

The synthesis was carried out according to the general procedure described above from indene (500 mg; 4.3 mmol) and 5-methylthiophene-2-carbaldehyde (543 mg, 4.3 mmol) in 1% KOH–ethanol (18 mL) stirred at reflux for 72 h. The product was purified by column chromatography on silica gel using petroleum ether and dichloromethane (3 : 1) as eluents to afford the pure compound 2 as a yellow solid.

2: 298 mg (30%); mp 123–124 °C; ^1^H NMR (CDCl_3_, 600 MHz) *δ*/ppm: 7,61 (dd, *J* = 6.9 Hz; 1.4 Hz, 1H), 7,45 (s, 1H), 7,30(dd, *J* = 6.9 Hz; 1.4 Hz, 1H), 7.22–7.16 (m, 3H), 7,12 (d, *J* = 3.6 Hz, 1H), 6.97 (dd, *J* = 5.9, 1.1 Hz, 1H), 6.75–6.73 (m, 1H), 2,55 (s, 3H); ^13^C NMR (CDCl_3_; 150 MHz) *δ*/ppm: 144.8, 141.8, 138.8, 137.7, 136.0, 133.4, 132.3, 126.9, 126.1, 125.7, 124.9, 121.6, 121.1, 118.8, 15.8; HRMS (LC-Q/TOF) *m*/*z* [M + H]^+^ calculated for C_15_H_12_S ^+^ 224.0643, found 224.0649.

#### 3.3.3 (*E*)-2-((1*H*-Inden-1-ylidene)methyl)phenol (3)

The synthesis was carried out according to the general procedure described above from indene (2.0 g; 17.2 mmol) and salicylaldehyde (2.1 g, 17.2 mmol) in 1% KOH–ethanol (72 mL) stirred at reflux for 48 h. The product was purified by column chromatography on silica gel using petroleum ether and dichloromethane (3 : 1) as eluents to afford the pure compound 3 as a yellow solid.

3: 707 mg (19%); mp 130–132 °C; ^1^H NMR (CDCl_3_, 600 MHz) *δ*/ppm: 7.73 (dd, *J* = 6.3, 1.7 Hz, 1H), 7.63 (s, 1H), 7.49 (d, *J* = 8.0 Hz, 1H), 7.34–7.27 (m, 2H), 7.25–7.23 (m, 1H), 7.03–7.68 (m, 2H), 6.89–6.85 (m, 2H), 5.18 (s, 1H); ^13^C NMR (CDCl_3_, 150 MHz) *δ*/ppm: 153.9, 142.4, 141.3, 137.0, 134.7, 131.7, 130.0, 127.6, 126.2, 125.3, 124.0, 123.1, 121.1, 121.0, 119.5, 116.0; HRMS (LC-Q/TOF) *m*/*z* [M + H]^+^ calculated for C_16_H_13_O^+^ 221.0962, found 221.0961.

#### 3.3.4 (*E*)-4-((1*H*-Inden-1-ylidene)methyl)-*N*,*N*-dimethylaniline (4)

The synthesis was carried out according to the general procedure described above from indene (2.0 g; 17.2 mmol) and *para*-dimethylaminobenzaldehyde (2.6 g, 17.2 mmol) in 1% KOH–ethanol (72 mL) stirred at reflux for 48 h. The product was purified by column chromatography on silica gel using petroleum ether and dichloromethane (3 : 1) as eluents to afford the pure compound 4 as yellow solid.

4: 1.9 g (45%); mp 141–142 °C; ^1^H NMR (CDCl_3_, 600 MHz) *δ*/ppm: 7.70–7.68 (m, 1H), 7.59 (d, *J* = 8.9 Hz, 2H), 7.43 (s, 1H), 7.33–7.32 (m, 1H), 7.21–7.19 (m, 2H), 7.15 (d, *J* = 5.5 Hz, 1H), 6.98 (dd, *J* = 5.5, 1.2 Hz, 1H), 6.75 (d, *J* = 8.9 Hz, 2H), 3.04 (s, 6H); ^13^C NMR (CDCl_3_, 150 MHz) *δ*/ppm: 150.5, 141.4, 138.1, 135.7, 133.2, 132.4, 132.0 (2C), 131.5, 129.8, 126.4, 126.0, 124.6, 120.8, 118.6, 112.1, 40.2 (2C); HRMS (LC-Q/TOF) *m*/*z* [M + H]^+^ calculated for C_18_H_18_N^+^ 248.1436, found 248.1434.

#### 3.3.5 (*E*)-3-((1*H*-inden-1-ylidene)methyl)pyridine (5)

The synthesis was carried out according to the general procedure described above from indene (500 mg; 4.3 mmol) and pyridine-3-carbaldehyde (461 mg, 4.3 mmol) in 1% KOH–ethanol (18 mL) stirred at reflux for 48 h. The product was purified by column chromatography on silica gel using petroleum ether and dichloromethane (3 : 1) as eluents to afford the pure compound 5 as a yellow solid.

5: 41 mg (5%); mp 132–133 °C; ^1^H NMR (CDCl_3_, 300 MHz) *δ*/ppm: 8.83 (d, *J* = 1.7 Hz, 1H), 8.57 (dd, *J* = 8.6, 1.5 Hz, 1H), 7.90 (dt, *J* = 8.1, 1.5 Hz, 1H), 7.70 (d, *J* = 6.4 Hz, 1H), 7.42 (s, 1H), 7.33–7.27 (m, 3H), 7.25–7.24 (m, 1H), 7.06 (dd, *J* = 5.5, 0.9 Hz, 1H), 6.94 (d, *J* = 5.5 Hz, 1H); ^13^C NMR (CDCl_3_, 75 MHz) *δ*/ppm: 151.0, 149.0, 142.1, 136.8, 135.9, 128.2, 125.6, 125.4, 124.4, 123.5, 121.2, 121.0, 119.4, some quarter C-atoms were not detected; HRMS (LC-Q/TOF) *m*/*z* [M + H]^+^ calculated for C_15_H_12_N^+^ 206.0968, found 206.0964.

### 3.4. General procedure for synthesis of indenes 6–9

A solution of previously synthesized indene 1–4 in dry THF (5 mL) was dropwise added to a suspension of lithium aluminum hydride (2 eq.) in dry THF at 0 °C, cooled by ice. The reaction mixture was stirred under the atmosphere of nitrogen at room temperature (rt) for 2 h. At the end of the reaction, the reaction mixture was diluted with diethyl ether (10 mL) and quenched with water (120 µL), 4 M aqueous solution of NaOH (120 µL) and water (360 µL) at 0 °C. The solution was filtered, extracted with diethyl ether (3 × 10 mL) and dried over anhydrous MgSO_4_. After evaporation, the residue was purified on a silica gel column with petroleum ether and dichloromethane (4 : 1) as eluents to isolate products 6–9.

#### 3.4.1 2-((1*H*-Inden-3-yl)methyl)-1*H*-pyrrole (6)^2^

The synthesis was carried out according to the general procedure described above from solution of compound 1 (307 mg; 1.6 mmol) in THF (8 mL) and suspension of lithium aluminum hydride (121 mg; 3.2 mmol) in dry THF (3 mL) stirred at rt for 2 h. The product was purified by column chromatography on silica gel using petroleum ether and dichloromethane (4 : 1) as eluents to afford the pure compound 6 white solid.

6: 22 mg (8.4%); mp 90–91 °C; ^1^H NMR (CDCl_3_, 600 MHz) *δ*/ppm: 7.93 (s, 1H), 7.46 (d, *J* = 7.2 Hz, 1H), 7.32 (d, *J* = 7.2 Hz, 1H), 7.25 (dt, *J* = 7.2, 1.2 Hz, 1H), 7.20 (dt, *J* = 7.2, 1.2 Hz, 1H), 6.66–6.64 (m, 1H), 6.28–6.25 (m, 1H), 6,15 (dd, *J* = 5.8, 2.9 *Hz*, 1H), 6.08–6.06 (m, 1H), 3.92 (s, 2H), 3.36 (d, *J* = 1.6 Hz, 2H); ^13^C NMR (CDCl_3_, 150 MHz) *δ*/ppm: 144.8 (s), 144.5 (s), 142.0 (s), 130.1 (d), 129.0 (s), 126.2 (d), 124.8 (d), 123.8 (d), 119.4 (d), 116.7 (d), 108.4 (d), 106.3 (d), 37.7 (t), 29.7 (t); HRMS (LC-Q/TOF) *m*/*z* [M + H]^+^ calculated for C_14_H_14_N^+^ 196.1119, found 196.1121.

#### 3.4.2 2-((1*H*-Inden-3-yl)methyl)-5-methylthiophene (7)

The synthesis was carried out according to the general procedure described above from solution of compound 2 (346 mg; 1.5 mmol) in THF (8 mL) and suspension of lithium aluminum hydride (117 mg; 3.0 mmol) in dry THF (3 mL) stirred at rt for 2 h. The product was purified by column chromatography on silica gel using petroleum ether and dichloromethane (4 : 1) as eluents to afford the pure compound 7 as white solid.

7: 58 mg (10%); mp 112–113 °C; ^1^H NMR (CDCl_3_, 600 MHz) *δ*/ppm: 7.45 (d, *J* = 7.6 Hz, 1H), 7,34 (d, *J* = 7.6 Hz, 1H), 7.26 (dt, *J* = 7.6, 0.7 *Hz*, 1H), 7,19 (dt, *J* = 7.6, 1.1 Hz, 1H), 6.65 (d, *J* = 3.2 Hz, 1H), 6.56–6.54 (m, 1H), 6.29–6.27 (m, 1H), 4.01 (s, 2H), 3.35 (d, *J* = 1.9 Hz, 2H), 2.41 (s, 3H); ^13^C NMR (CDCl_3_, 150 MHz) *δ*/ppm: 144.7 (s), 144.5 (s), 142.9 (s), 139.7 (s), 138.1 (s), 129.9 (d), 126.1 (d), 125.1 (d), 124.8 (d), 124.7 (d), 123.8 (d), 119.3 (d), 37.7 (t), 30.4 (q), 28.9 (t); HRMS (LC-Q/TOF) *m*/*z* [M + H]^+^ calculated for C_15_H_15_S^+^ 227.0889, found 227.0886.

#### 3.4.3 2-((1*H*-Inden-3-yl)methyl)phenol (8)

The synthesis was carried out according to the general procedure described above from solution of compound 3 (376 mg; 1.7 mmol) in THF (10 mL) and suspension of lithium aluminum hydride (130 mg; 3.4 mmol) in dry THF (3.5 mL) stirred at rt for 2 h. The product was purified by column chromatography on silica gel using petroleum ether and dichloromethane (4 : 1) as eluents to afford the pure compound 8 as white solid.

8: 131 mg (35%); mp 124–125 °C; ^1^H NMR (CDCl_3_, 600 MHz) *δ*/ppm: 7.47 (d, *J* = 7.3 Hz, 1H), 7.39 (d, *J* = 7.3 Hz, 1H), 7.29 (t, *J* = 7.3 Hz, 1H), 7.21–7.20 (m, 2H), 7.16 (td, *J* = 7.4, 1.1 Hz, 1H), 6.91 (td, *J* = 7.4, 1.1 Hz, 1H), 6.85 (dd, *J* = 7.4, 1.1 Hz, 1H), 6.23–6.12 (m, 1H), 4.93 (s, 1H), 3.92 (d, *J* = 1.9 Hz, 2H), 3.37 (d, *J* = 2.1 Hz, 2H); ^13^C NMR (CDCl_3_, 150 MHz) *δ*/ppm: 154.12, 144.66, 144.60, 142.22, 130.86, 129.97, 127.94, 126.15, 124.95, 124.89, 123.81, 120.95, 119.43, 37.75, 29.31; HRMS (LC-Q/TOF) *m*/*z* [M + H]^+^ calculated for C_16_H_15_O^+^ 223.1116, found 223.1117.

#### 3.4.4 4-((1*H*-Inden-3-yl)methyl)-*N*,*N*-dimethylaniline (9)

The synthesis was carried out according to the general procedure described above from solution of compound 4 (500 mg; 2.0 mmol) in THF (18 mL) and suspension of lithium aluminum hydride (153 mg; 4.0 mmol) in dry THF (3.5 mL) stirred at rt for 2 h. The product was purified by column chromatography on silica gel using petroleum ether and dichloromethane (4 : 1) as eluents to afford the pure compound 9 as white solid.

9: 219 mg (44%); mp 135–136 °C; ^1^H NMR (CDCl_3_, 600 MHz) *δ*/ppm: 7.40 (d, *J* = 7.3 Hz, 1H), 7.29 (d, *J* = 7.3 Hz, 1H), 7.21 (t, *J* = 7.3 Hz, 1H), 7.16–7.13 (m, 1H), 7.12 (d, *J* = 8.7 Hz, 2H), 6.66 (d, *J* = 8.7 Hz, 2H), 6.11–6.07 (m, 1H), 3.78 (d, *J* = 1.4 Hz, 2H), 3.29 (d, *J* = 1.9 Hz, 2H), 2.86 (s, 6H); ^13^C NMR (CDCl_3_, 150 MHz) *δ*/ppm: 148.9, 145.3, 144.6, 144.2, 129.6 (2C), 129.6 (2C), 127.9, 126.0, 124.5, 123.7, 119.4, 113.2, 41.1, 37.7, 33.4; HRMS (LC-Q/TOF) *m*/*z* [M + H]^+^ calculated for C_18_H_20_N^+^ 250.1589, found 250.1590.

### 3.5. General procedure for synthesis of indenes 10–13

Indole (2 mmol) and corresponding aldehyde (1 mmol) were added to the stirred acetonitrile solution (6 mL) of Cu(NO_3_)_2_·3H_2_O (10 mol%) and mixed for 1.5–16 h at room temperature. After completion of the reaction, the crude product was extracted with diethyl ether, dried over anhydrous MgSO_4_ and concentrated under reduced pressure to furnish the crude product, which was further purified by silica gel chromatography using petroleum ether and ethyl–acetate (9 : 1) as eluents, to afford products 10–13, respectively.

#### 3.5.1 3,3′*-*(phenylmethylene)bis(1*H*-indole) (10)^40^

The synthesis was carried out according to the general procedure described above from indole (469 mg; 4 mmol) and benzaldehyde (212 mg; 2 mmol) in suspension of Cu(NO_3_)_2_·3H_2_O (48 mg; 0.02 mmol) in acetonitrile (6 mL) stirred at rt for 1.5 hours. The product was purified by column chromatography on silica gel using petroleum ether and dichloromethane (9 : 1) as eluents to afford the pure compound 10 as red solid.

10: 268 mg (42%); mp 145–146 °C; ^1^H NMR (CDCl_3_, 300 MHz) *δ*/ppm: 7.92 (s, 2H), 7.43–7.27 (m, 8H), 7.24–7.11 (m, 3H), 7.00 (t, *J* = 7.5 Hz, 2H), 6.67 (m, 2H), 5.89 (s, 1H); ^13^C NMR (CDCl_3_, 75 MHz) *δ*/ppm: 144.0, 136.7 (2C), 128.7 (2C), 128.2 (2C), 127.1 (2C), 126.1, 123.6 (2C), 121.9 (2C), 119.9 (2C), 119.8 (2C), 119.2 (2C), 111.0 (2C), 40.2; HRMS (LC-Q/TOF) *m*/*z* [M + H]^+^ calculated for C_23_H_19_N_2_^+^ 323.1546, found 323.1543.

#### 3.5.2 3,3′*-*(Ethane-1,1-diyl)bis(1*H*-indole) (11)^41^

The synthesis was carried out according to the general procedure described above from indole (469 mg; 4 mmol) and acetaldehyde (88 mg; 2 mmol) in the suspension of Cu(NO_3_)_2_·3H_2_O (48 mg; 0.02 mmol) in acetonitrile (6 mL) stirred at rt for 1.5 hours. The product was purified by column chromatography on silica gel using petroleum ether and dichloromethane (9 : 1) as eluents to afford the pure compound 11 as red solid.

11: 294 mg (57%); mp 145–148 °C; ^1^H NMR (CDCl_3_, 600 MHz) *δ*/ppm: 7.85 (s, 2H), 7.59 (d, *J* = 7.9 Hz, 2H), 7.35 (d, *J* = 7.9 Hz, 2H), 7.18 (dt, *J* = 8.1, 1.2 Hz, 2H), 7.06 (dt, *J* = 8.1, 1.2 Hz, 2H), 6.93–6.90 (m, 2H), 4.69 (q, *J* = 7.1 Hz, 1H), 1.88–1.78 (m, 3H); ^13^C NMR (CDCl_3_, 150 MHz) *δ*/ppm: 136.6 (2C), 126.9 (2C), 121.8 (2C), 121.7 (2C), 121.2 (2C), 119.7 (2C), 119.0 (2C), 111.0 (2C), 28.2, 21.7.

#### 3.5.3 3,3′*-*((5-Methylthiophen-2-yl)methylene)bis(1*H*-indole) (12):^5^

The synthesis was carried out according to the general procedure described above from indole (469 mg; 0.4 mmol) and 5-methylthiophene-2-carbaldehyde (252 mg; 0.2 mmol) in suspension of Cu(NO_3_)_2_·3H_2_O (48 mg; 0.02 mmol) in acetonitrile (6 mL) stirred at rt for 16 h. The product was purified by column chromatography on silica gel using petroleum ether and dichloromethane (9 : 1) as eluents to afford the pure compound 12 as red solid.

12: 377 mg (55%); mp 147–148 °C; ^1^H NMR (CDCl_3_, 600 MHz) *δ*/ppm: 7.94 (s, 2H), 7.48 (d, *J* = 8.1 Hz, 2H), 7.36 (d, *J* = 8.1 Hz, 2H), 7.17 (dt, *J* = 8.1, 1.1 Hz, 2H), 7.03 (dt, *J* = 8.1, 1.1 Hz, 2H), 6.90–6.86 (m, 2H), 6.67 (d, *J* = 3.3 Hz, 1H), 6.56–6.52 (m, 1H), 6.07 (s, 1H), 2.40 (s, 3H); ^13^C NMR (CDCl_3_, 150 MHz) *δ*/ppm: 146.0, 137.9, 136.6 (2C), 126.8 (2C), 124.8, 124.4, 123.1 (2C), 122.0 (2C), 119.8 (2C), 119.7, 119.3 (2C), 111.1 (2C), 35.5, 15.4; HRMS (LC-Q/TOF) *m*/*z* [M + H]^+^ calculated for C_22_H_19_N_2_S^+^ 343.1263, found 343.1263.

#### 3.5.4 3,3′*-*(Pyridin-3-ylmethylene)bis(1*H*-indole) (13)^42^

The synthesis was carried out according to the general procedure described above from indole (469 mg; 0.4 mmol) and pyridine-3-carbaldehyde (214 mg; 0.2 mmol) in suspension of Cu(NO_3_)_2_·3H_2_O (48 mg; 0.02 mmol) in acetonitrile (6 mL) stirred at rt for 16 hours. The product was purified by column chromatography on silica gel using petroleum ether and dichloromethane (9 : 1) as eluents to afford the pure compound 13 as red solid.

13: 76 mg (12%); mp 141–142 °C; ^1^H NMR (CDCl_3_, 600 MHz) *δ*/ppm: 8.67–8.66 (m, 1H), 8.47 (dd, *J* = 4.8, 1.6 Hz, 1H), 8.08 (s, 2H), 7.60 (dt, *J* = 7.8, 1.8 Hz, 1H), 7.37 (d, *J* = 8.8 Hz, 4H), 7.20 (d, *J* = 4.8 Hz, 1H), 7.17 (td, *J* = 8.1, 1.1 Hz, 2H), 7.01 (td, *J* = 8.1, 1.1 Hz, 2H), 6.67–6.66 (m, 2H), 5.92 (s, 1H); ^13^C NMR (CDCl_3_, 150 MHz) *δ*/ppm: 150.3, 147.45, 139.5, 136.7 (2C), 136.2, 126.7 (2C), 123.7 (2C), 123.3, 122.2 (2C), 119.7 (2C), 119.5 (2C), 118.5 (2C), 111.2 (2C), 37.8; HRMS (LC-Q/TOF) *m*/*z* [M + H]^+^ calculated for C_22_H_18_N_3_^+^ 324.1497, found 324.1495.

### 3.6. Computational study

The crystal structure of *E. coli* DNA Gyrase B (PDB ID: 6F86) and 14α-sterol demethylase (CYP51) (PDB ID: 1EA1) were obtained from the Protein Data Bank in PDB format. Receptor preparation was performed using UCSF ChimeraX 1.8 software^43^ where the native ligand and water molecules were removed. Additionally, AutoDockTools (MGLTools 1.5.7.)^44^ was used to add polar hydrogens and Kollman charges to the target protein. The prepared protein was saved in PDBQT format for *in silico* simulations. The active site of *E. coli* DNA gyrase B was identified using the X-ray crystal structure 6F86 with a resolution of 1.90 Å based on the position of the native inhibitor. The ATP-binding pocket of DNA gyrase B was found to consist of Asn46, Asp73, Val43, Glu50, Gly77, Thr165, Ile78, Val120, Ala47 and Arg136.^45^ The active site of 14α-sterol demethylase is determined to be located within a cavity of the enzyme, containing a central heme group.^46^

Geometry optimization of the selected ligands and standard drugs were obtained at the SMD/M06-2X/6-31G(d,p) level of theory using Gaussian program package.^47^ Frequency calculations were performed under the harmonic approximation on all the optimized structures at the same level of theory with no scaling in order to confirm that the structures correspond to the true minima meaning that no imaginary frequencies were present, as well as to extract thermal Gibbs free energy corrections. The solvent effect was implemented using the SMD solvation model,^48^ with the solvent relative permittivity set to *ε* = 35.69 (acetonitrile). The lowest energy conformation for each compound was selected and saved in.mol2 format. Furthermore, a torsional tree was determined for each compound, and files were saved in PDBQT format using AutoDockTools.

AutoDock 4.2.6 software package was used for docking the potential inhibitors of DNA gyrase B (PDB ID: 6F86) and of 14α-sterol demethylase (PDB ID: 1EA1). The grid box was constructed with dimensions 126 × 126 × 126 in the *x*, *y*, and *z* directions, with a grid point spacing of 0.475 Å, and centered at *x* = 67.364 Å, *y* = 31.991 Å, *z* = 54.406 Å to cover the entire protein for docking to the DNA gyrase B. In the case of targeted enzyme 14α-sterol demethylase simulations the grid box was constructed with dimensions 126 × 46 × 125 in the *x*, *y*, and *z* directions, with a grid point spacing of 0.420 Å, and centered at *x* = −16.794 Å, *y* = −7.007 Å and *z* = 62.781 Å. The Lamarckian genetic algorithm (LGA) was used to determine the globally optimized conformation. Docking simulations were carried out using a mutation rate of 0.02, a population size of 150, a crossover rate of 0.80, a maximum number of generation of 27 000, a maximum number of energy evaluations of 2 500 000 and 100 runs. The docking poses were clustered with RMSD less than 2.0 Å and the most populated cluster has been considered for further analysis. Protein–ligand interactions were visualized using BIOVIA Discovery Studio 2020 software.^49^

### 3.7. *In silico* prediction of pharmacokinetics

The structures of synthesized compounds 3, 5, 6, 8, 11 and 13 were converted to their canonical simplified molecular input line entry system (SMILES) and then submitted to SwissADME to predict their pharmacokinetic profile and drug-likeness.

## 4. Conclusions

In this study, indene and indole derivatives were successfully synthesized and characterized, in order to investigate their antimicrobial activity. Although some of them are known compounds, the antibacterial and antifungal activity was tested for the first time. Most of the compounds showed antifungal activity against *Candida lipolytica* 59 and *Aspergillus niger* 405 and antibacterial activity against *Bacillus subtilis* 3020, while none of the derivatives showed antibacterial activity against *Pseudomonas aeruginosa* 3011. The most optimal candidates for further structural modifications were phenolic (3), pyridine (5) and pyrrolic derivatives (6), as they showed both antibacterial and antifungal activity. The *in silico* investigations demonstrated that the most potent antimicrobial candidates exhibited favorable binding modes and binding site affinities towards DNA gyrase B and 14α-sterol demethylase, as well as favorable ADME profiles. Research into novel synthesized compounds can lead to the development of new drugs that represent an effective therapy against microbial resistance.

## Author contributions

Conceptualization, D. V., M. V. D. and I. D.; methodology, V. L. and M. Š. R.; software, I. D. and N. B.; validation, D. V., M. V. D. and I. D.; formal analysis, J. L. C. and V.L.; investigation, V. L., M. Š. R. and N. B.; resources, D. V. and M. V. D.; data curation, V. L., J. L. C., D. V. and I. D.; writing—original draft preparation, D. V. and I. D.; writing—review and editing, D. V., M. V. D. and I. D.; visualization, V. L., N. B. and I. D.; supervision, D. V. and I. D.; project administration, D. V.; funding acquisition, D. V. and M. V. D. All authors have read and agreed to the published version of the manuscript.

## Conflicts of interest

The authors declare no conflicts of interest.

## Supplementary Material

RA-016-D5RA08239K-s001

## Data Availability

The data supporting this article have been included as part of the Supplementary information (SI). Supplementary information: detailed experimental procedures including characterization data and spectra are given as supporting information. See DOI: https://doi.org/10.1039/d5ra08239k.
