# Supplementary material for: Indene and indole-based compounds as potential antimicrobial agents: synthesis, activity, docking studies and ADME analysis
Source: RSC Adv. 2026 Feb 23;16(12):10582–96. doi: 10.1039/d5ra08239k (PMC12928186; doi:10.1039/d5ra08239k)
Supplement: RA-016-D5RA08239K-s001 [file RA-016-D5RA08239K-s001.pdf]

Supplementary Information (SI) for RSC Advances.
This journal is © The Royal Society of Chemistry 2026

Supporting Information

for

Indene and indole-based compounds as potential antimicrobial agents: synthesis, activity, docking studies and ADME analysis

Vilma Lovrinčević,[1] Monika Šabić Runjavec,[2] Nikica Baričević,[1] Ines Despotović,[3] Jerome Le-Cunff,[4] Dragana Vuk*[1] and Marija Vuković Domanovac*[2]

[1]Department of Organic Chemistry, University of Zagreb Faculty of Chemical Engineering and Technology, Trg Marka Marulića 19, HR-10000 Zagreb, Croatia

[2]Department of Industrial Ecology, University of Zagreb Faculty of Chemical Engineering and Technology, Trg Marka Marulića 19, HR-10000 Zagreb, Croatia

[3]Division of Physical Chemistry, Ruđer Bošković Institute, Bijenička cesta 54, HR-10000 Zagreb, Croatia

[4]Xellia Ltd., Slavonska Avenija bb, 10000 Zagreb, Croatia

*Correspondening authors: dvuk@fkit.unizg.hr; mvukovic@fkit.unizg.hr

Content:

1. NMR spectra S2
2. Cartesian coordinates of optimized ligands docked into DNA gyrase B and 14α-sterol demethylase S15
3. Estimated free energies of binding obtained by molecular docking (Table S1, Table S2) S20

1. NMR spectra

^{1}H NMR ($CDCl_3$, 600 MHz) of compound **1**.

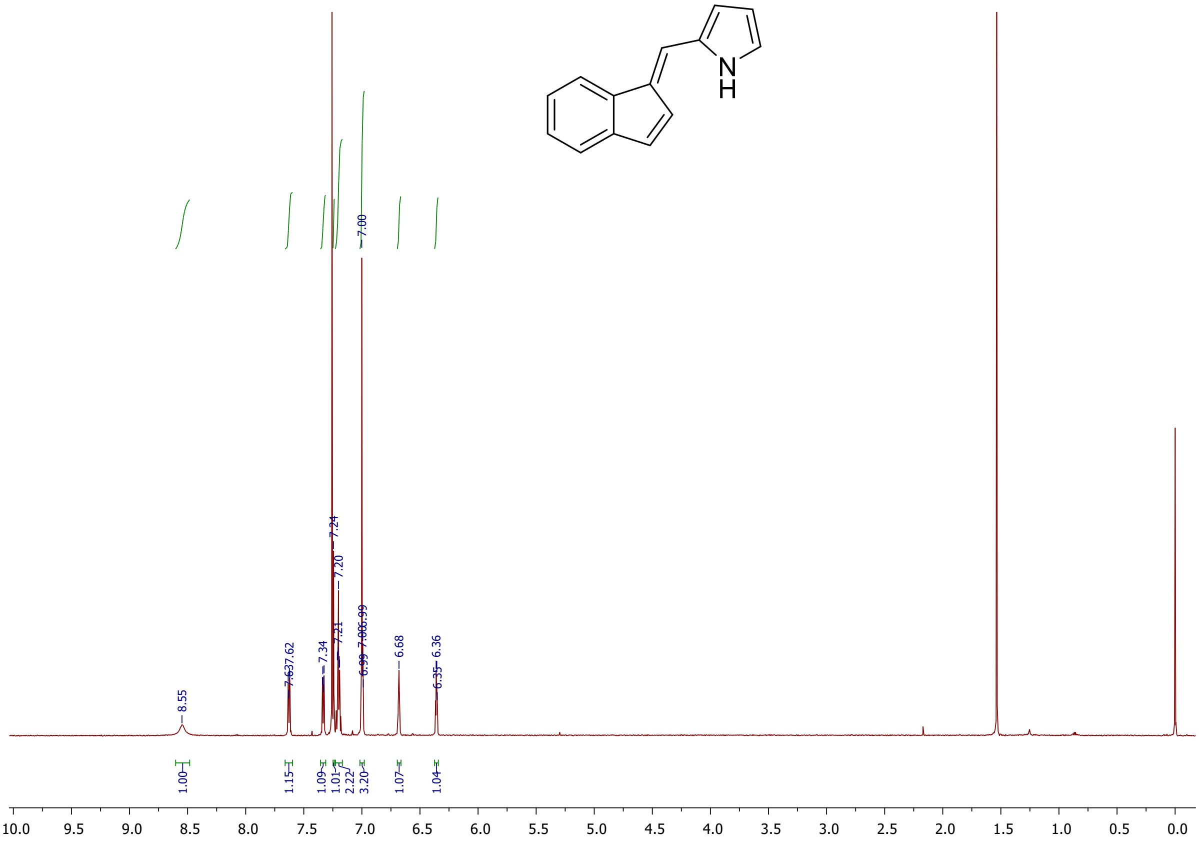

^{13}C NMR ($CDCl_3$, 150 MHz) of compound **1**.

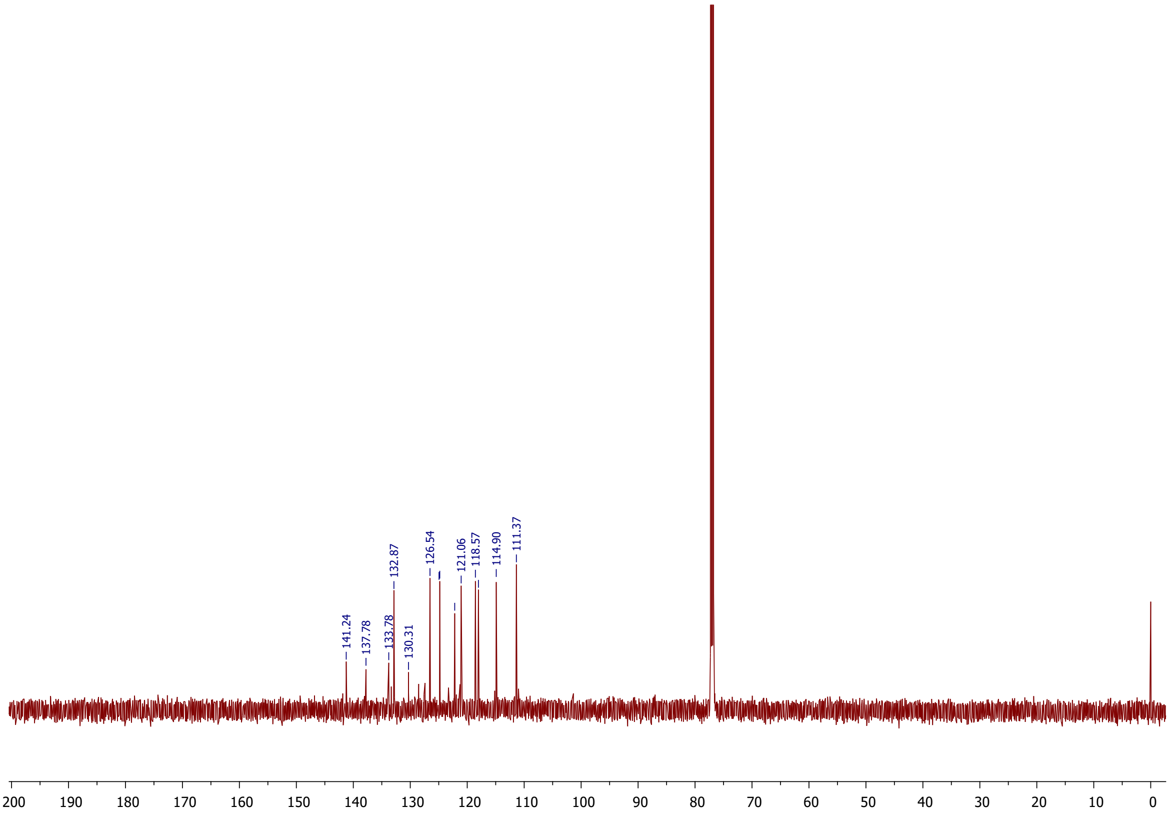

^{1}H NMR ($CDCl_3$, 600 MHz) of compound **2**.

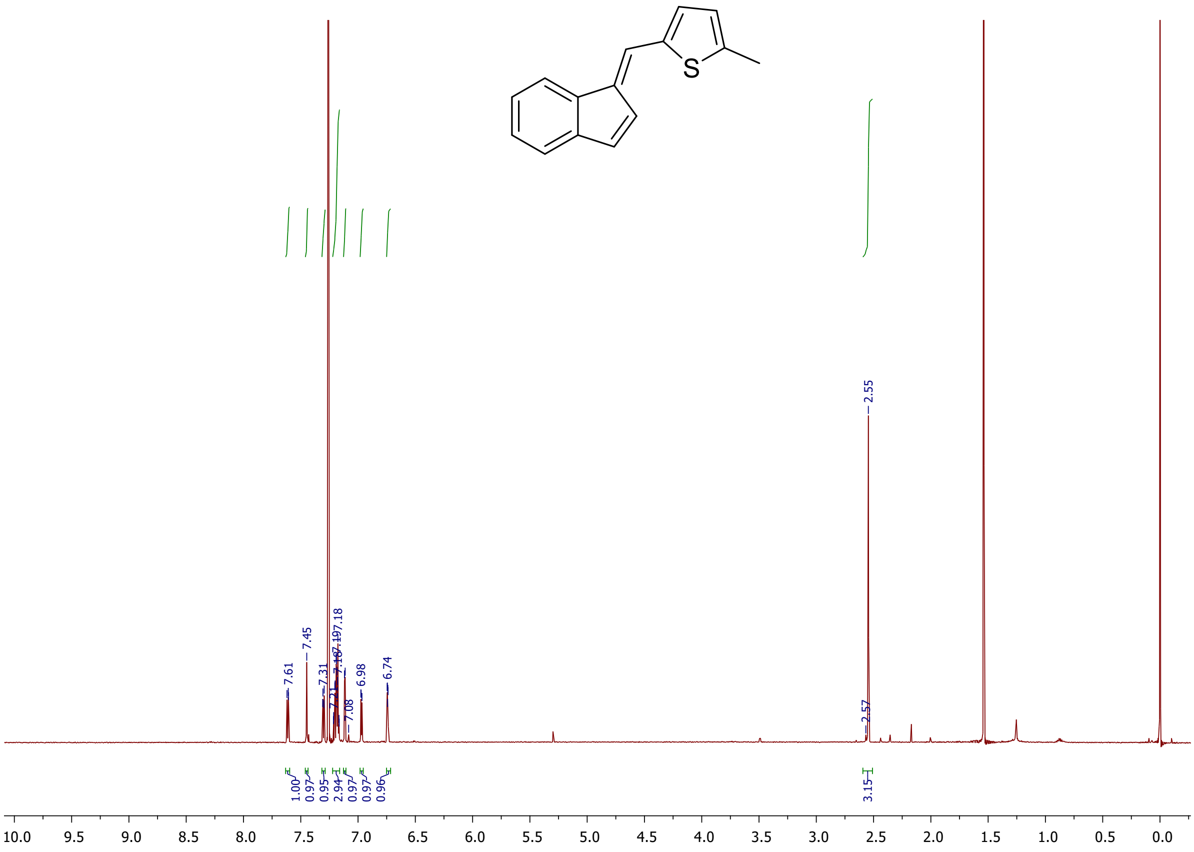

^{13}C NMR ($CDCl_3$, 150 MHz) of compound **2**.

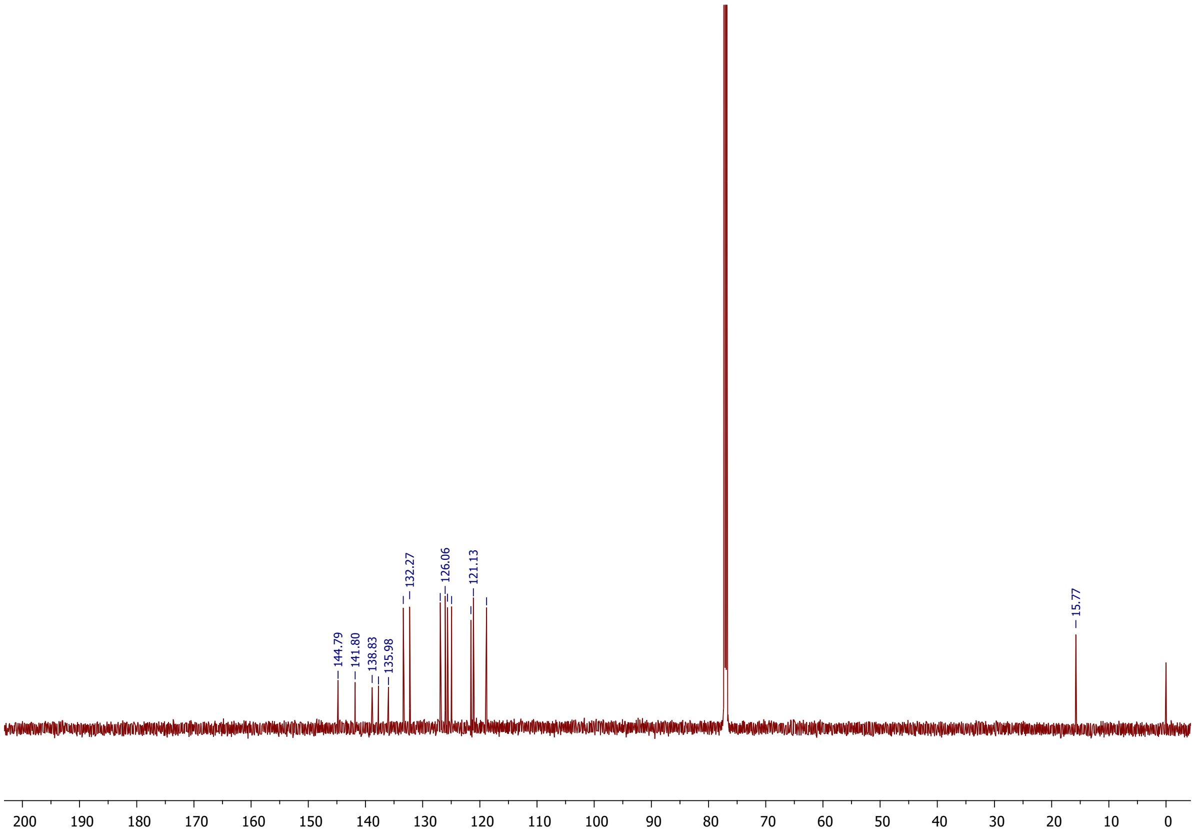

^{1}H NMR ($CDCl_3$, 300 MHz) of compound **3**.

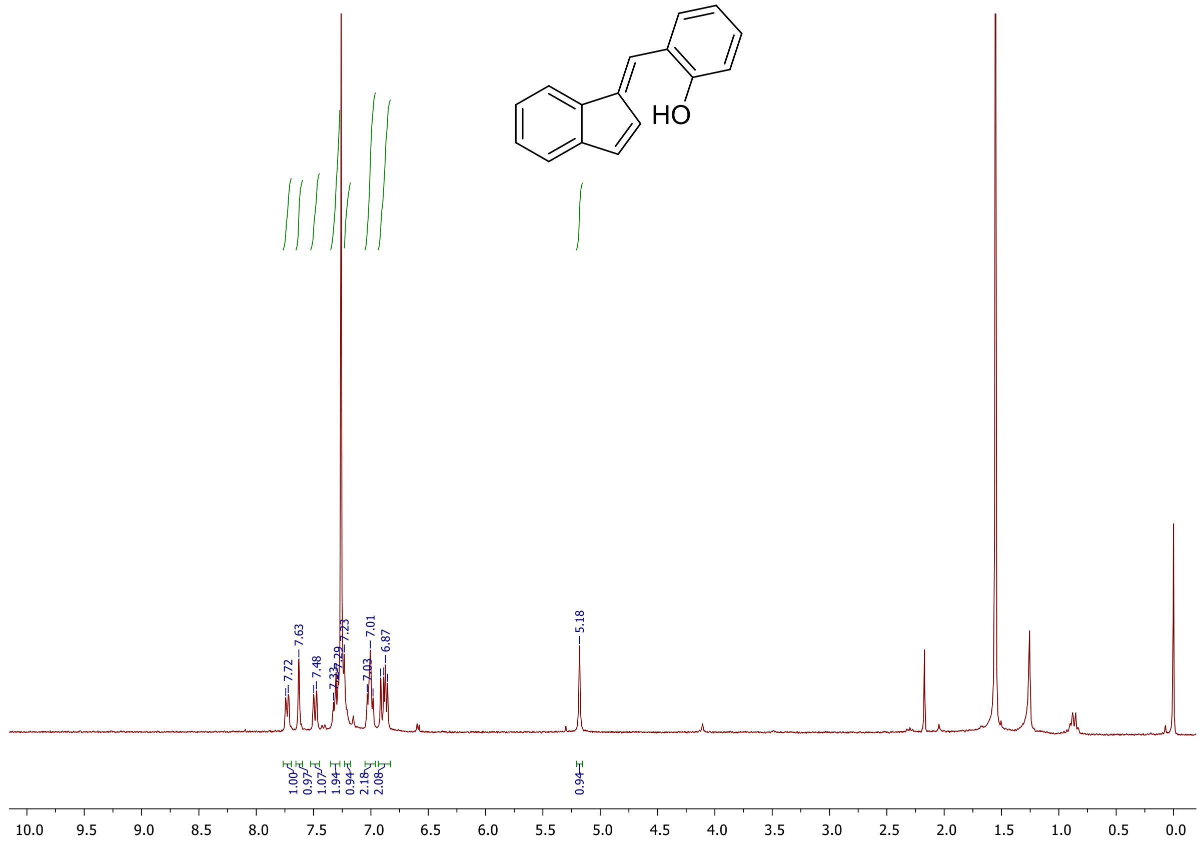

^{13}C NMR ($CDCl_3$, 150 MHz) of compound **3**.

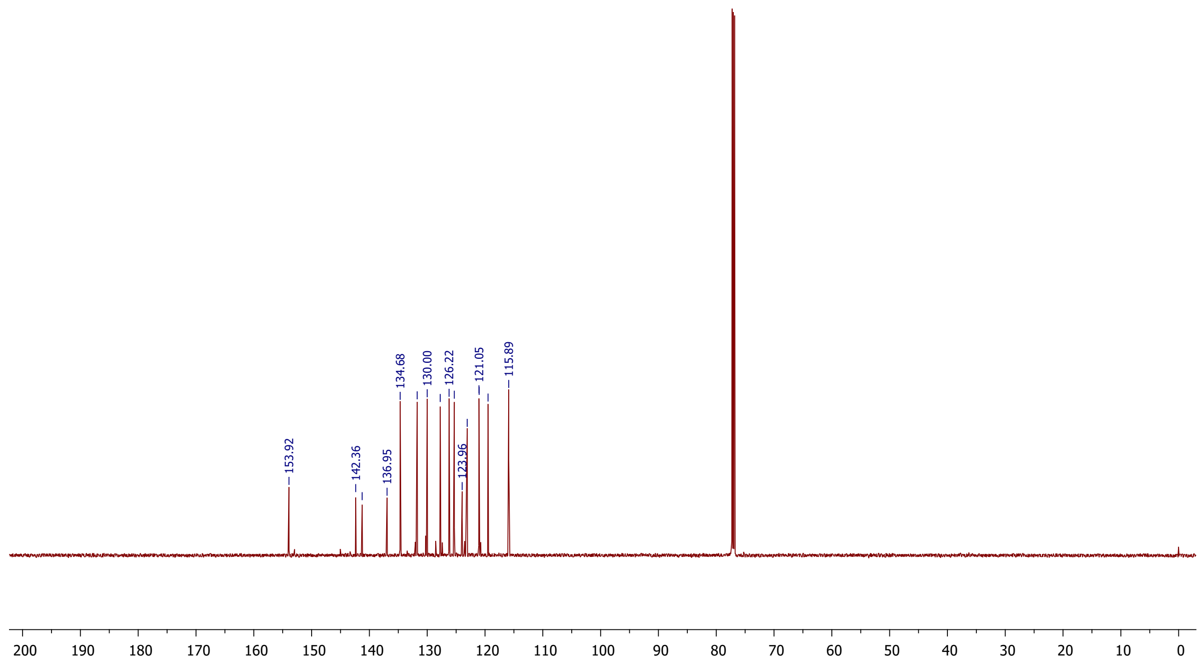

^{1}H NMR ($CDCl_3$, 600 MHz) of compound **4**.

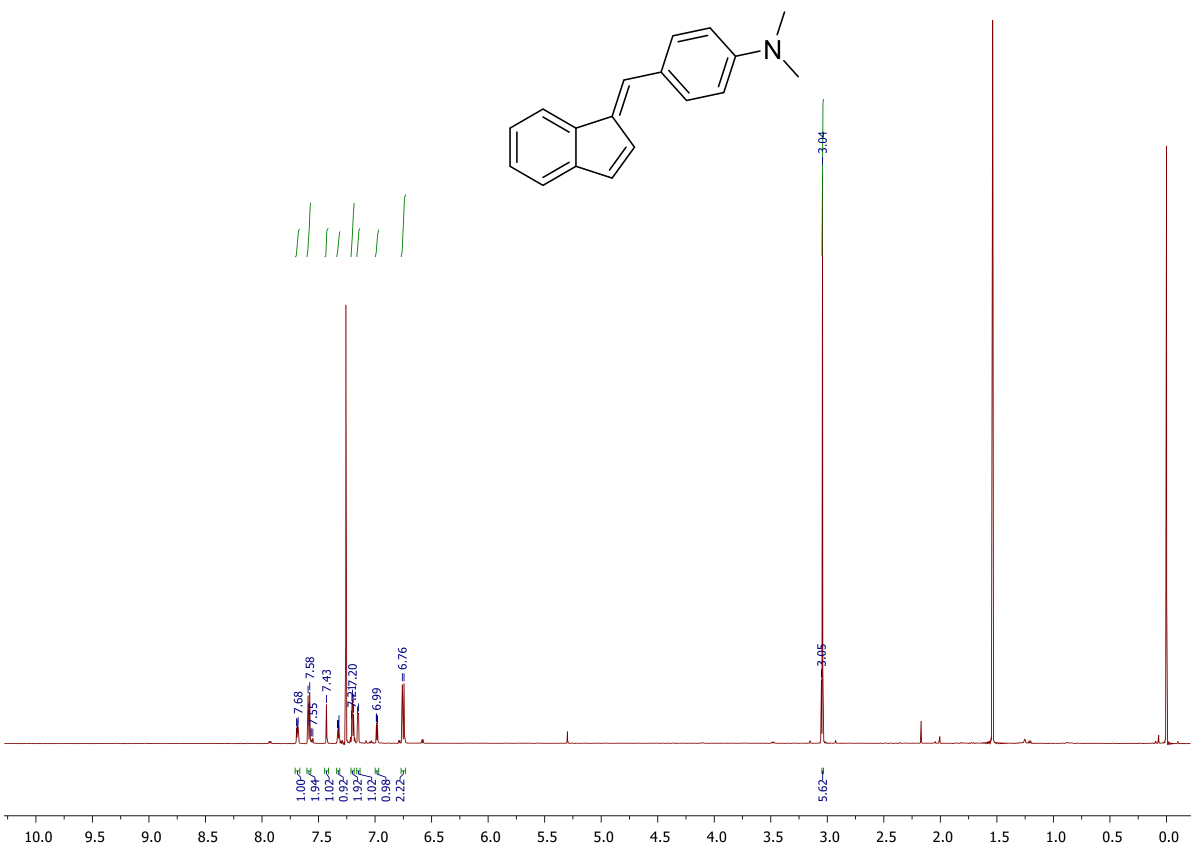

^{13}C NMR ($CDCl_3$, 150 MHz) of compound **4**.

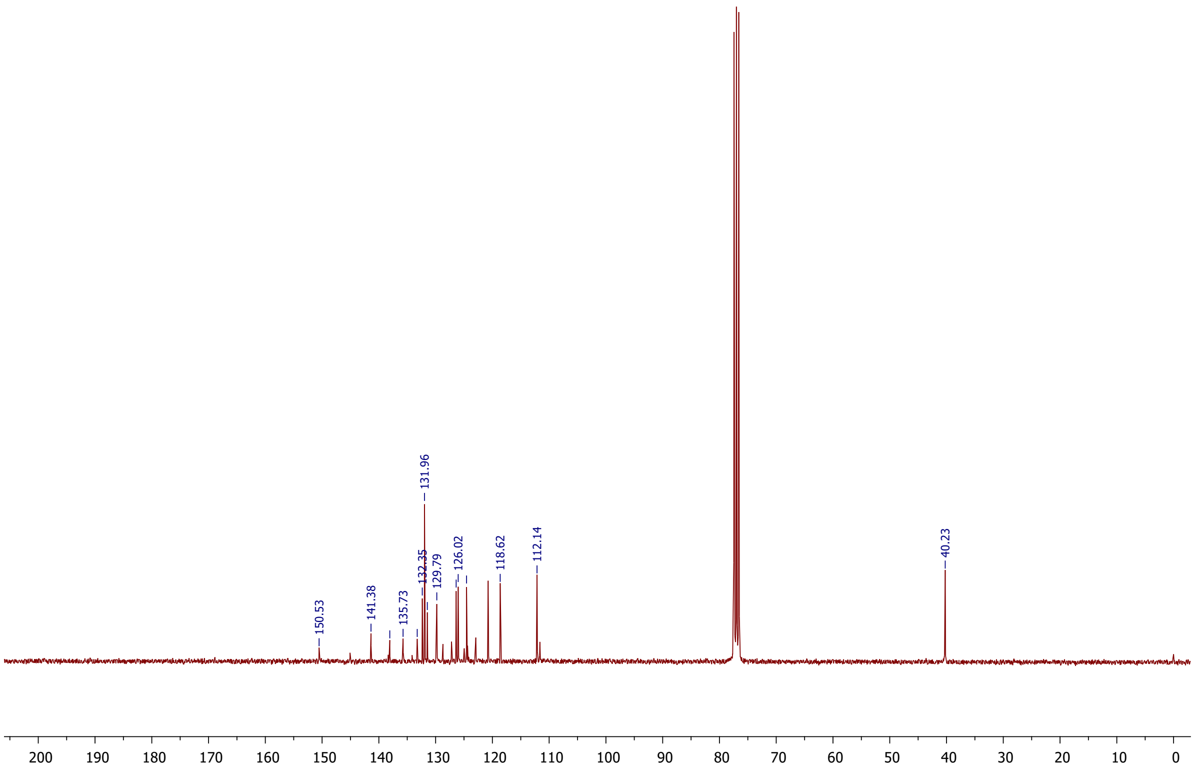

^{1}H NMR ($CDCl_3$, 300 MHz) of compound **5**.

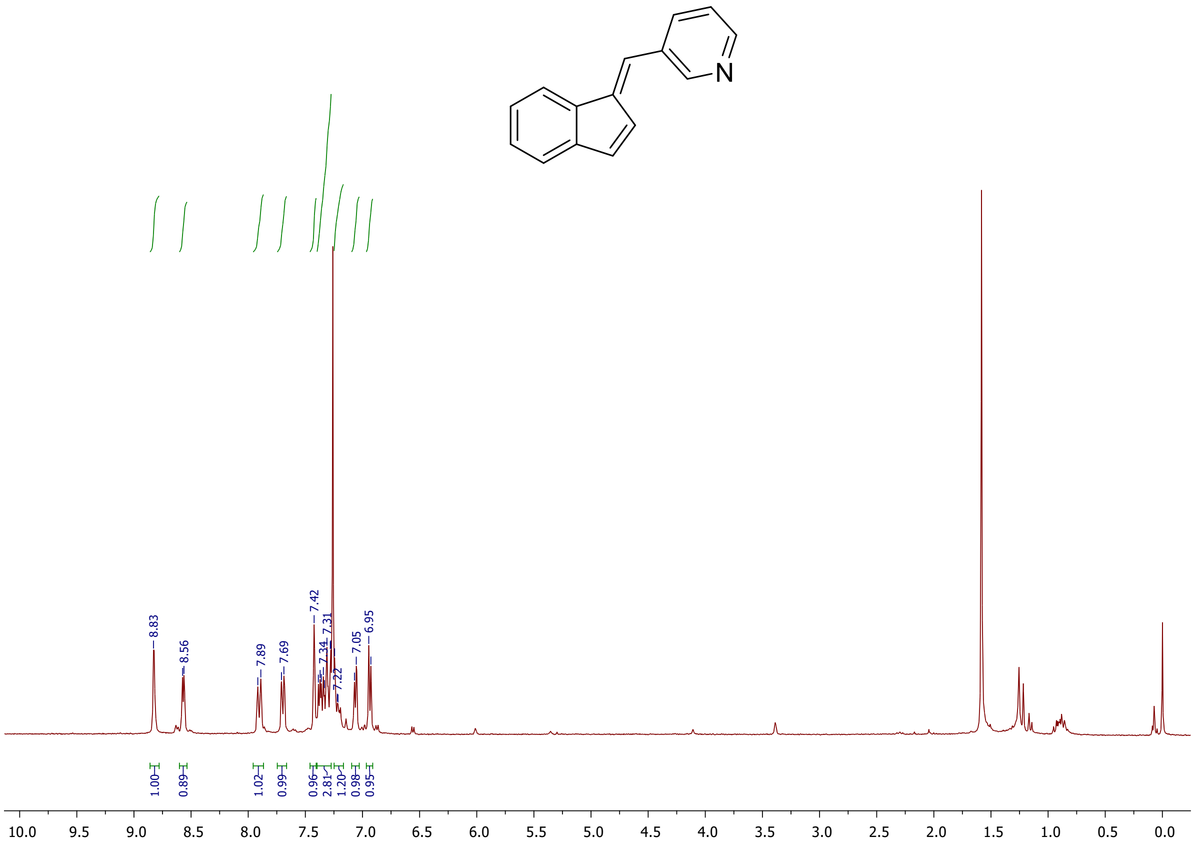

^{13}C NMR ($CDCl_3$, 75 MHz) of compound **5**.

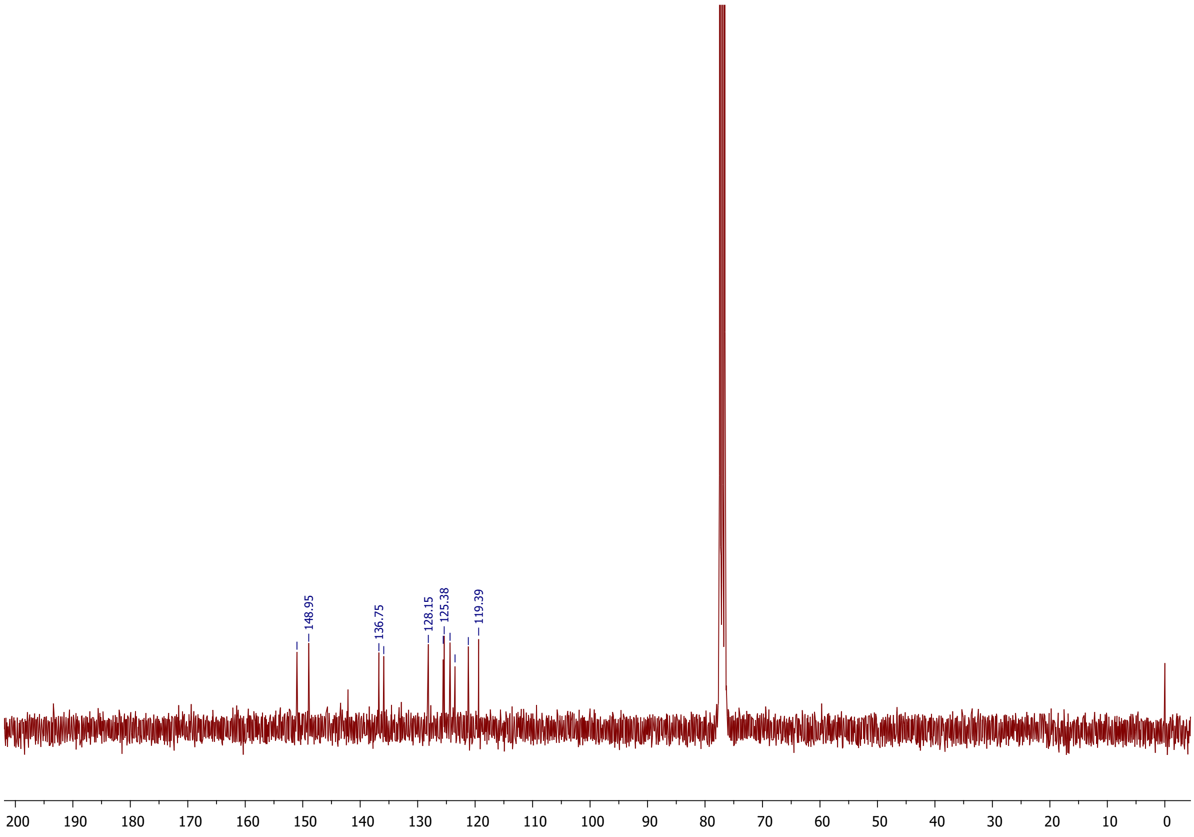

^{1}H NMR ($CDCl_3$, 600 MHz) of compound **6** (with 5% of compound **1**).

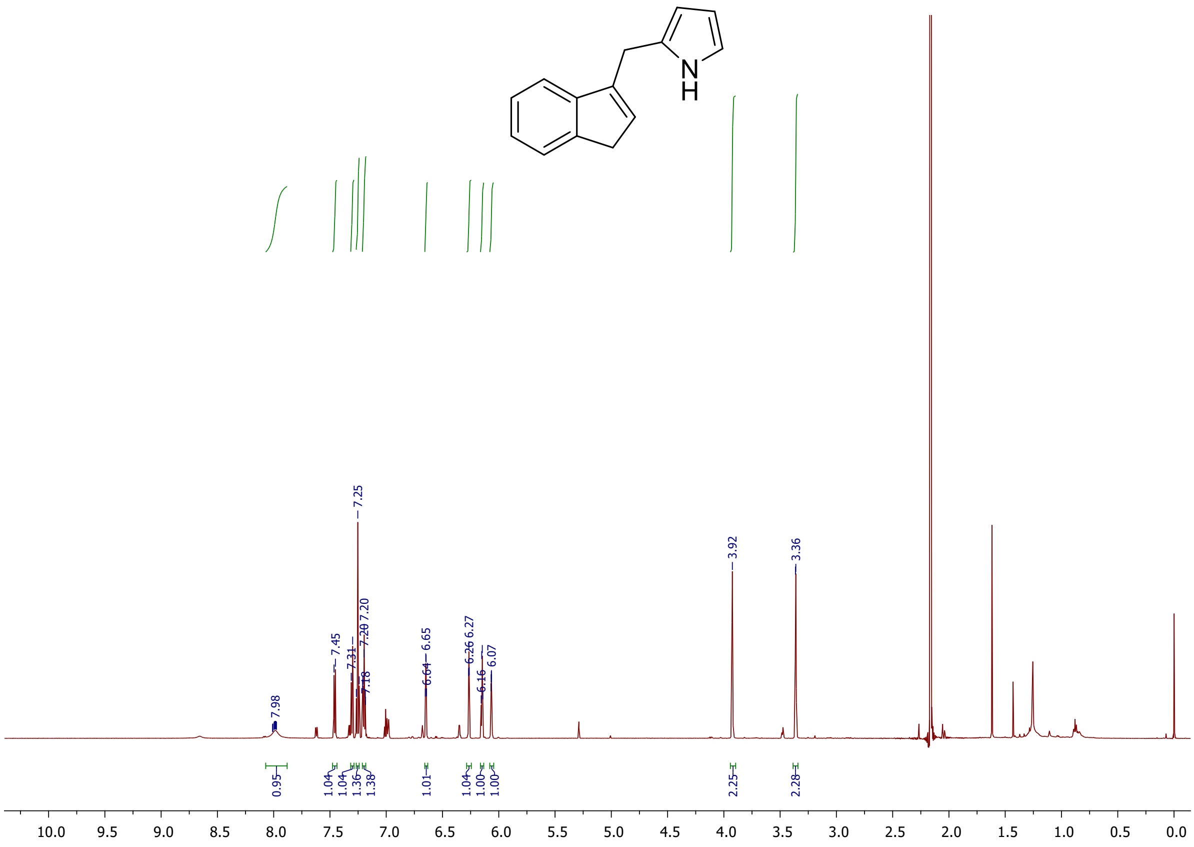

^{13}C NMR ($CDCl_3$, 150 MHz) of compound **6**(with 5% of compound **1**).

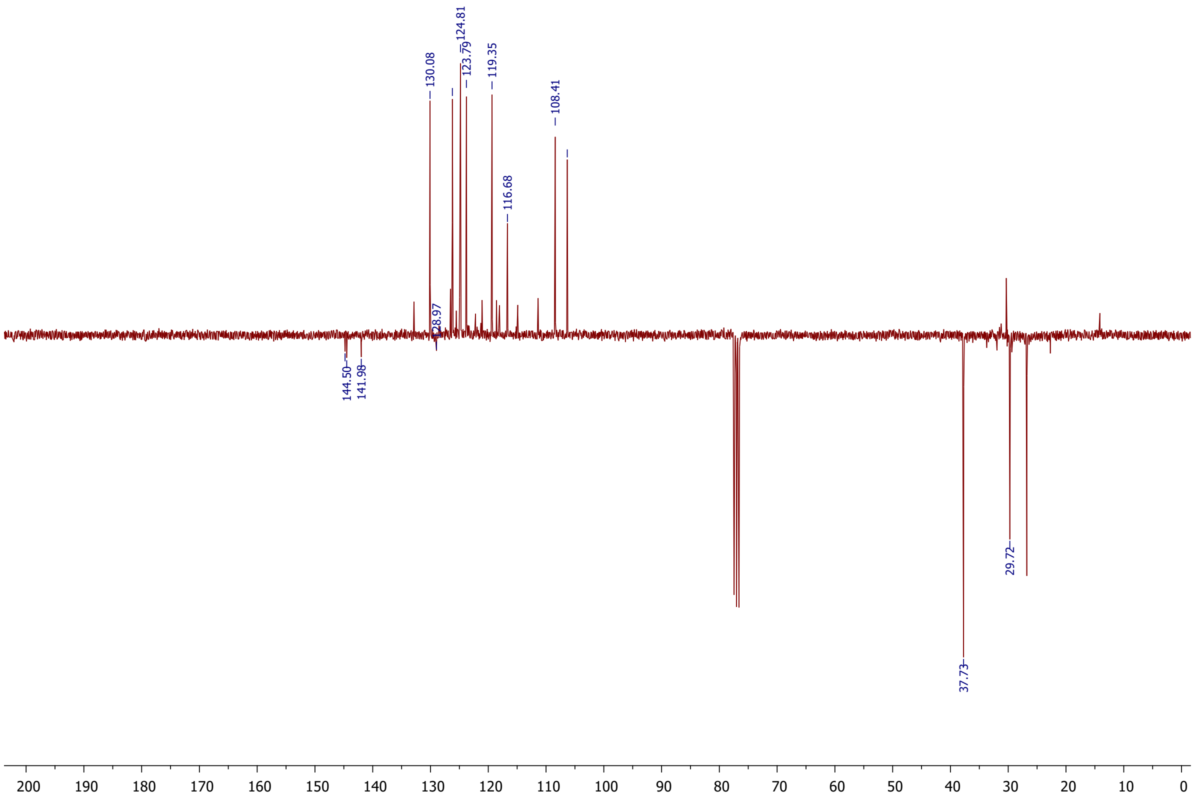

^{1}H NMR ($CDCl_3$, 600 MHz) of compound **7**.

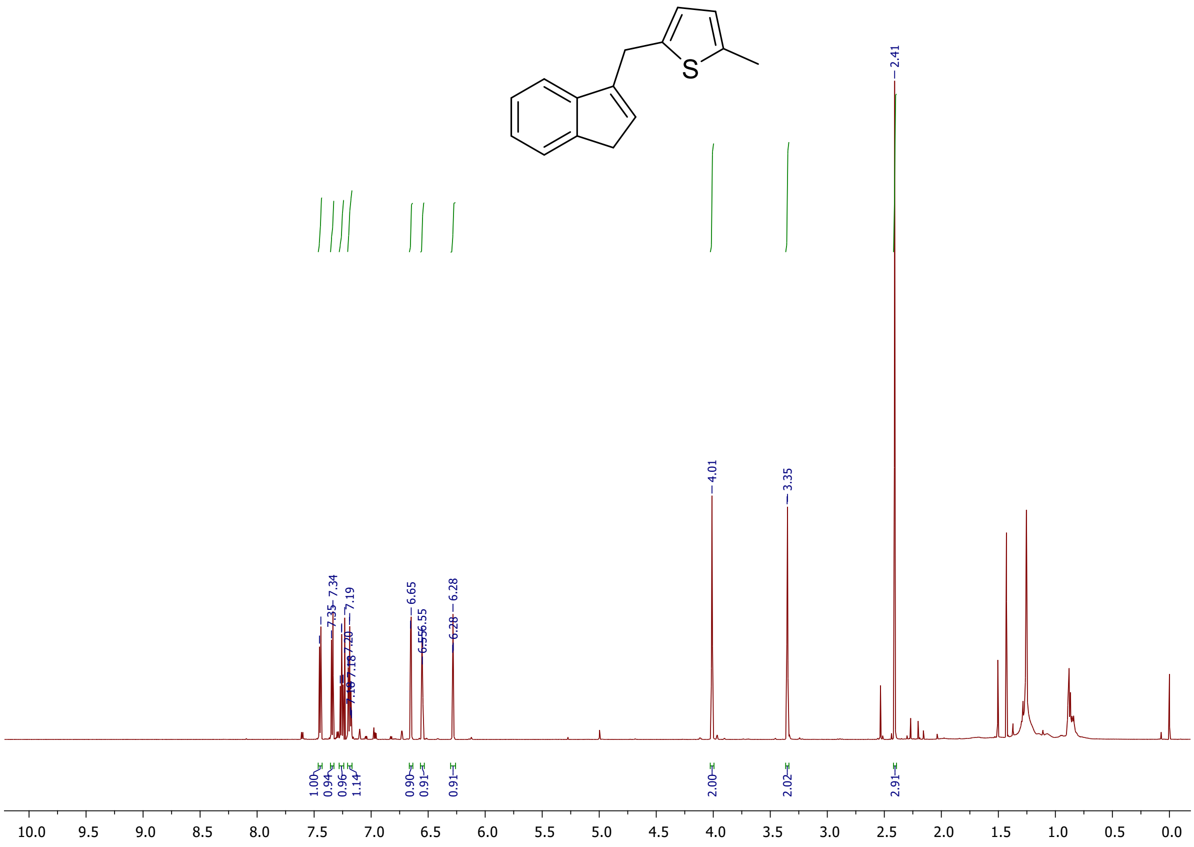

^{13}C NMR ($CDCl_3$, 150 MHz) of compound **7**.

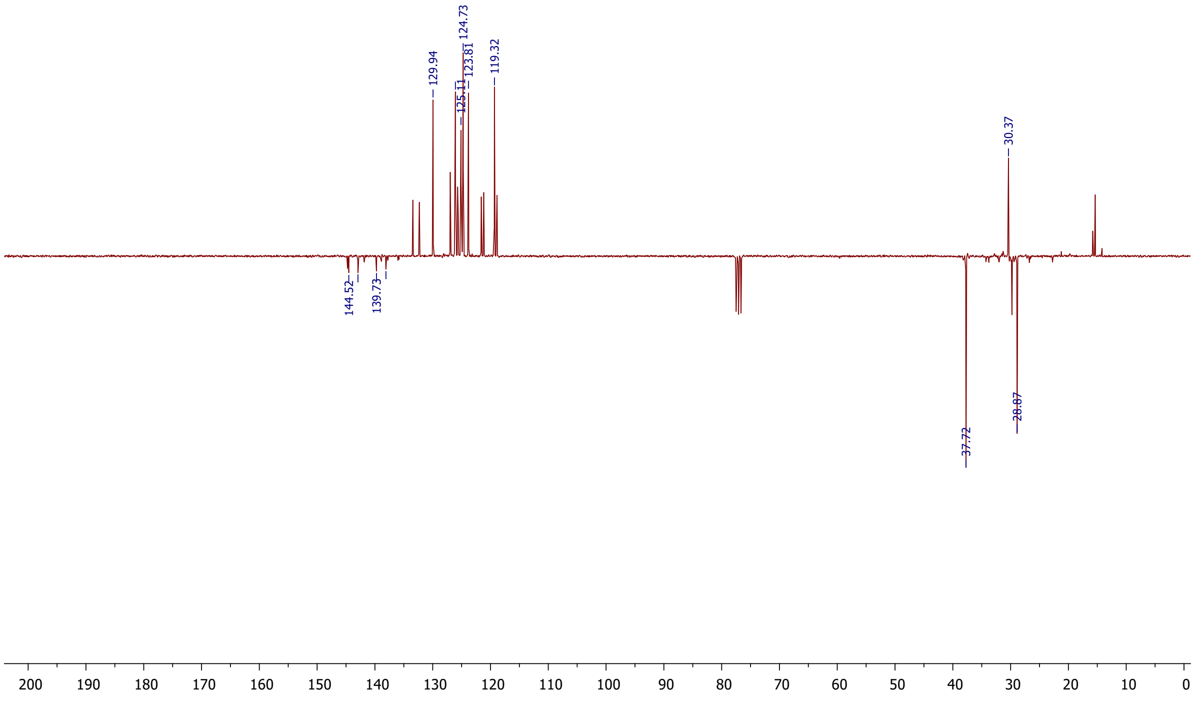

^{1}H NMR ($CDCl_3$, 600 MHz) of compound **8**.

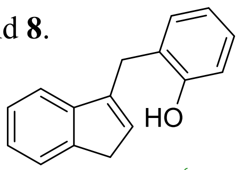

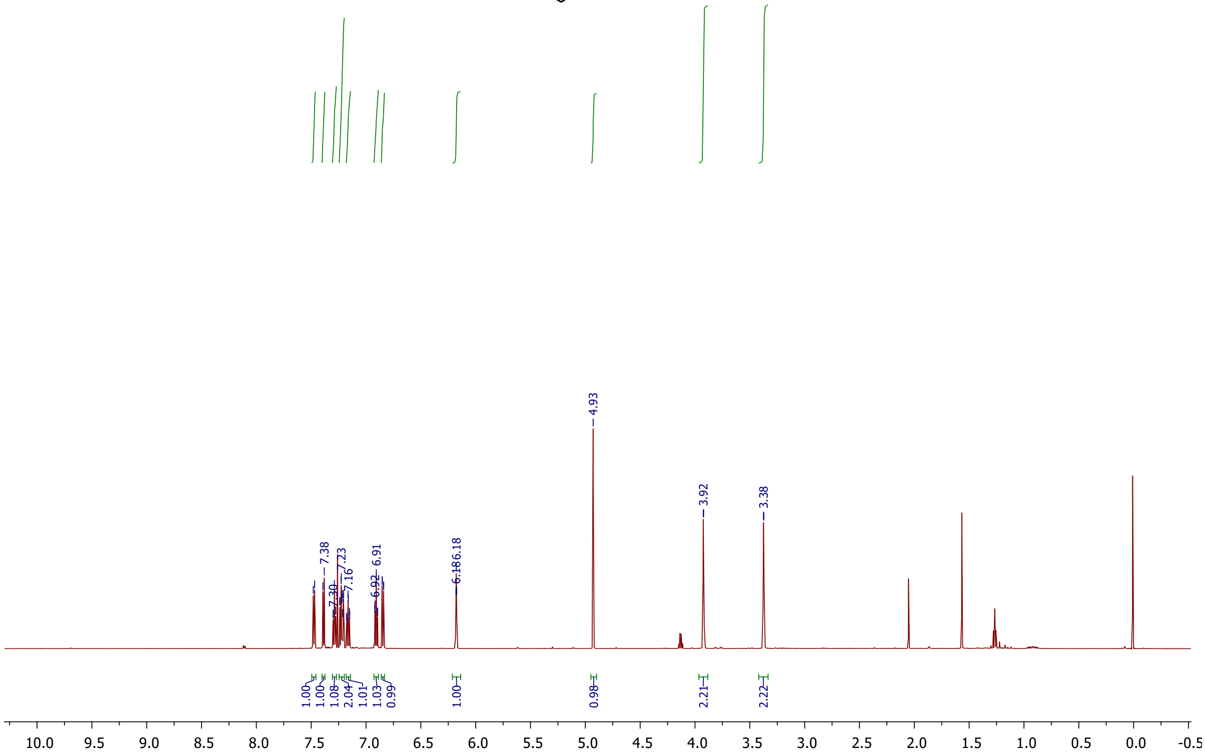

^{13}C NMR ($CDCl_3$, 150 MHz) of compound **8**.

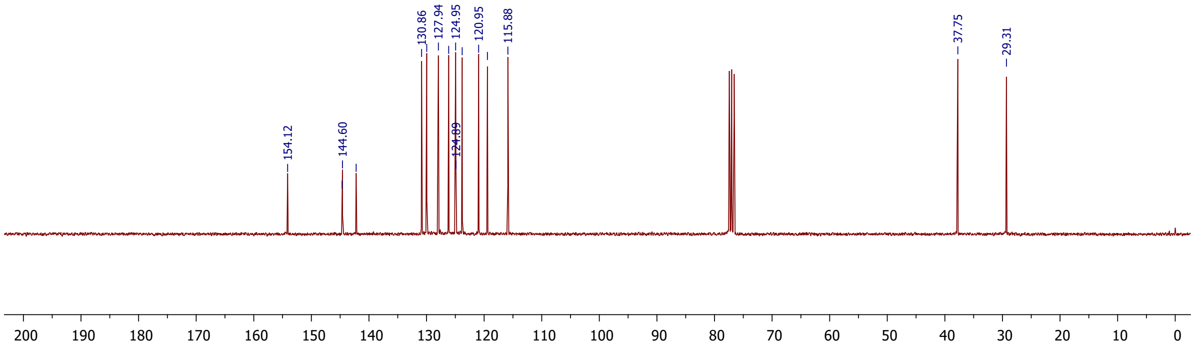

^{1}H NMR ($CDCl_3$, 600 MHz) of compound **9**.

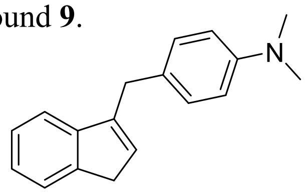

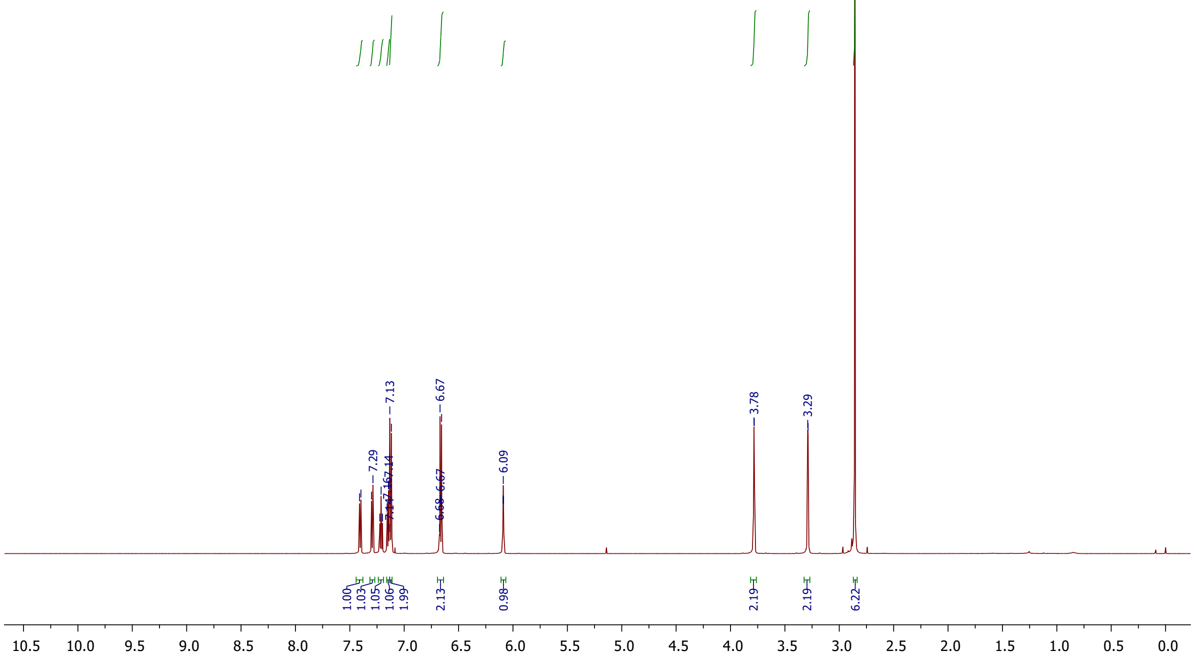

^{13}C NMR ($CDCl_3$, 150 MHz) of compound **9**.

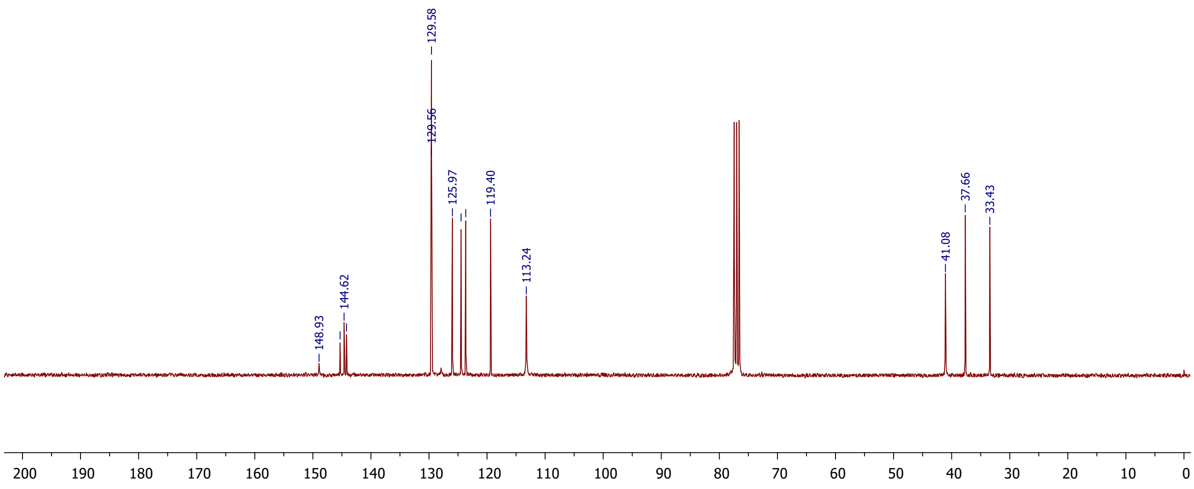

^{1}H NMR ($CDCl_3$, 300 MHz) of compound **10**.

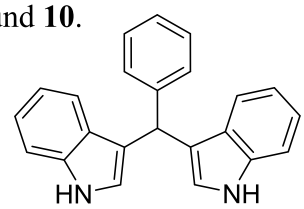

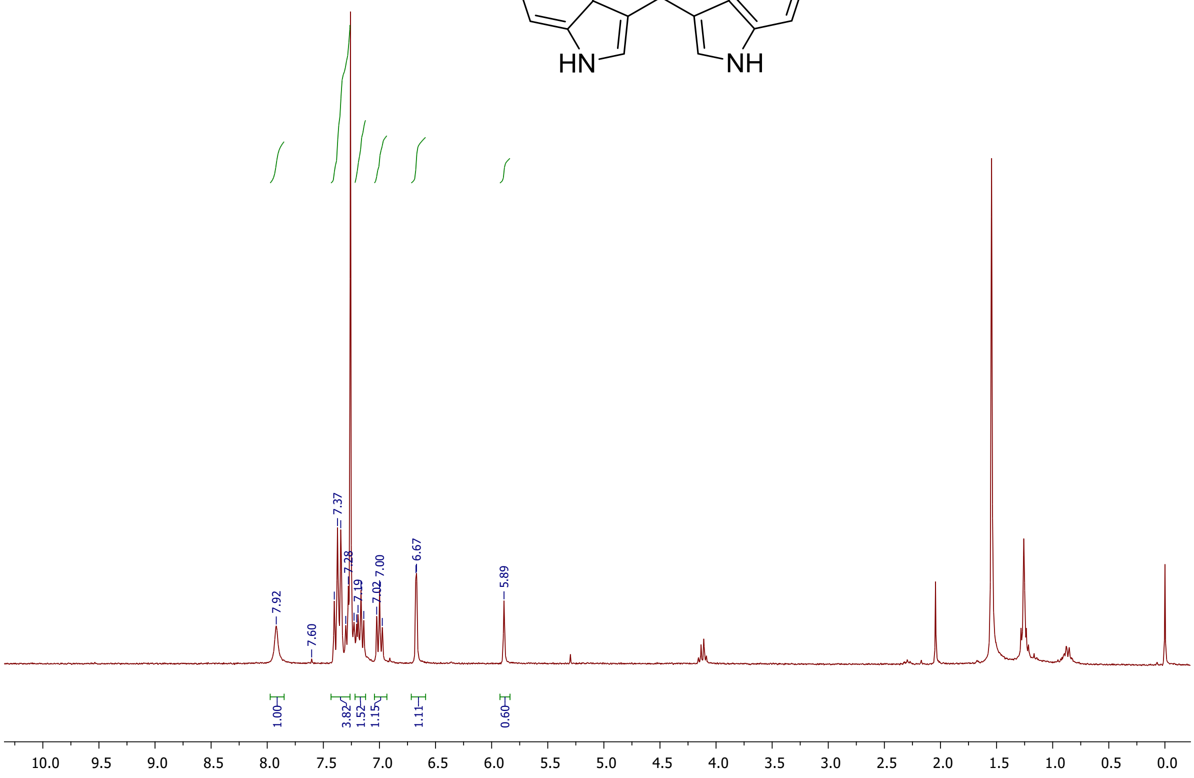

^{13}C NMR ($CDCl_3$, 75 MHz) of compound **10**.

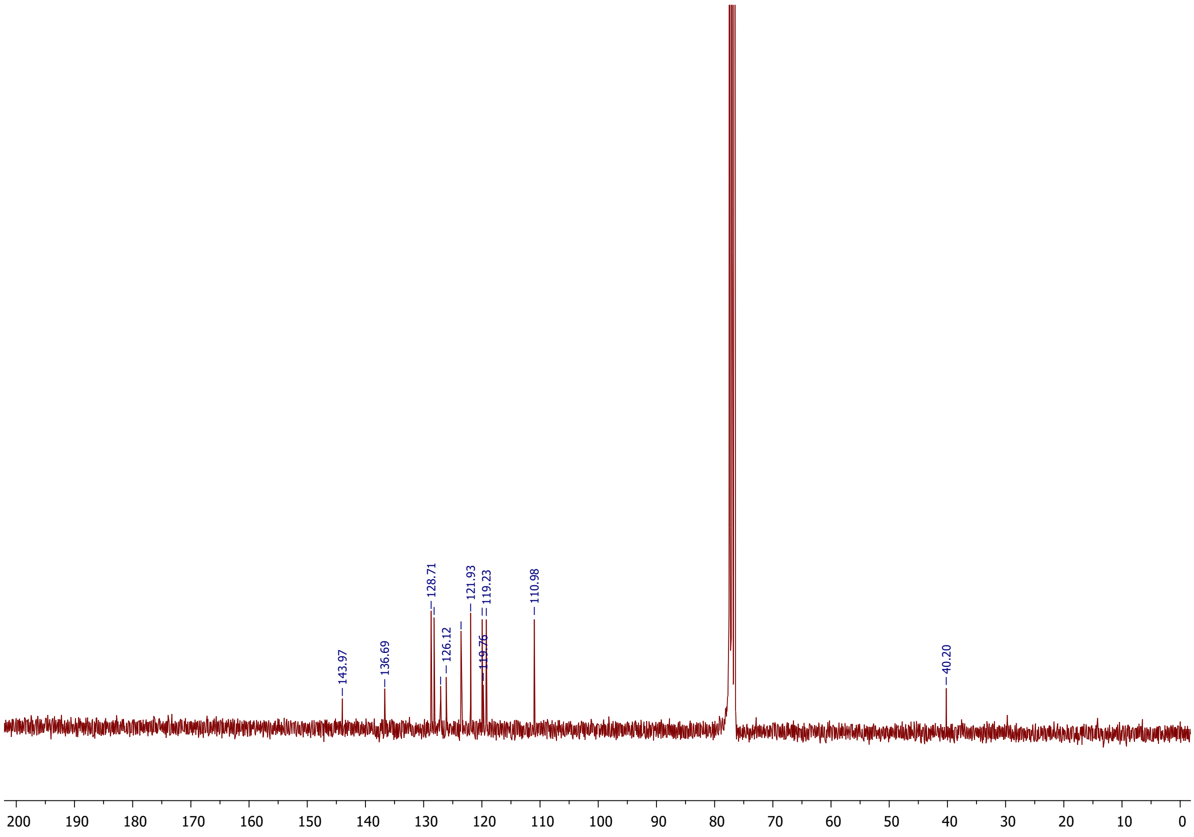

^{1}H NMR ($CDCl_3$, 600 MHz) of compound **11** (with 5% of stereoisomer).

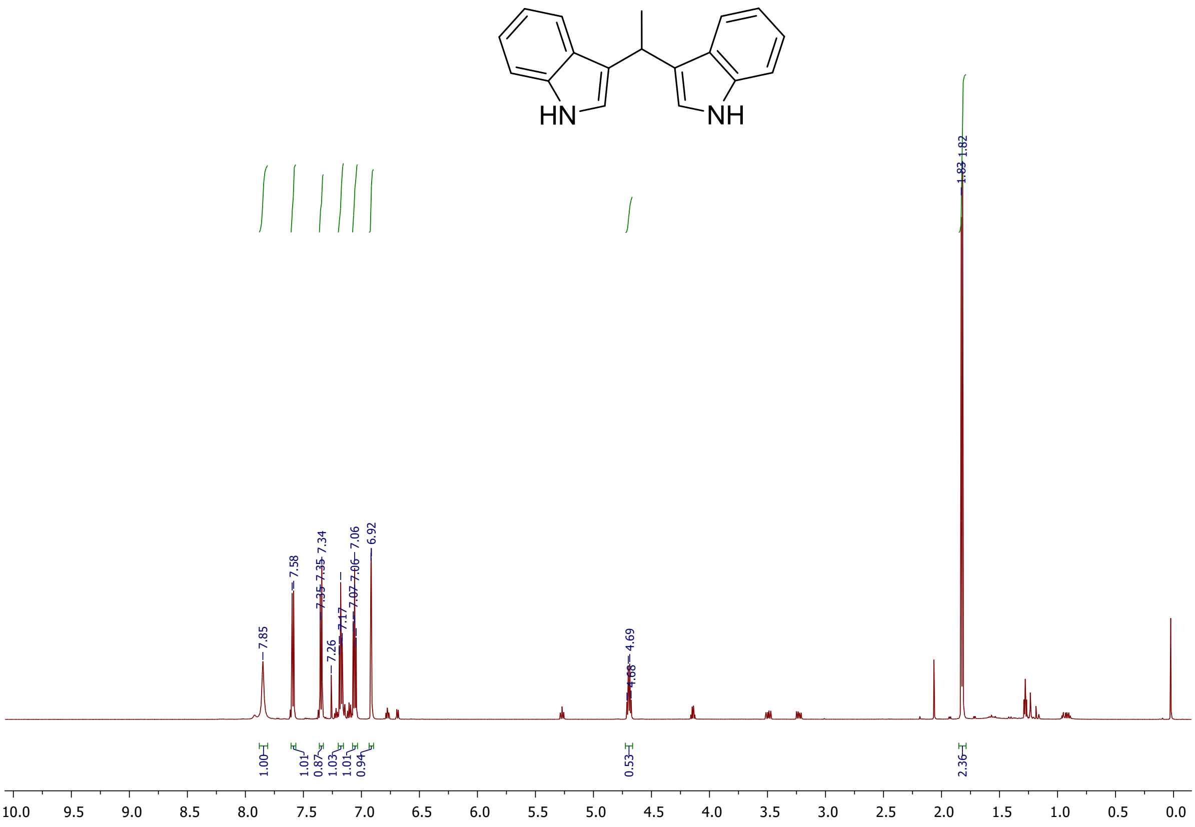

^{13}C NMR ($CDCl_3$, 150 MHz) of compound **11** (with 5% of stereoisomer).

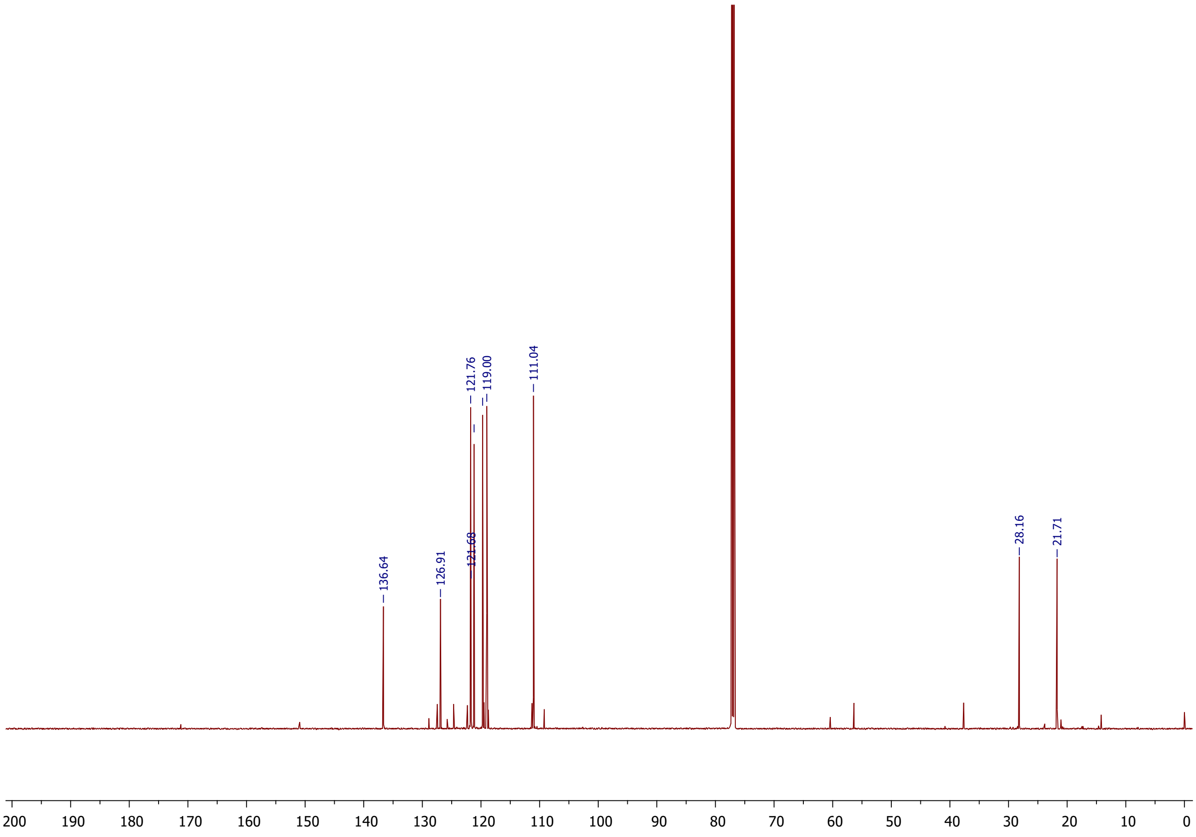

^{1}H NMR ($CDCl_3$, 600 MHz) of compound **12**.

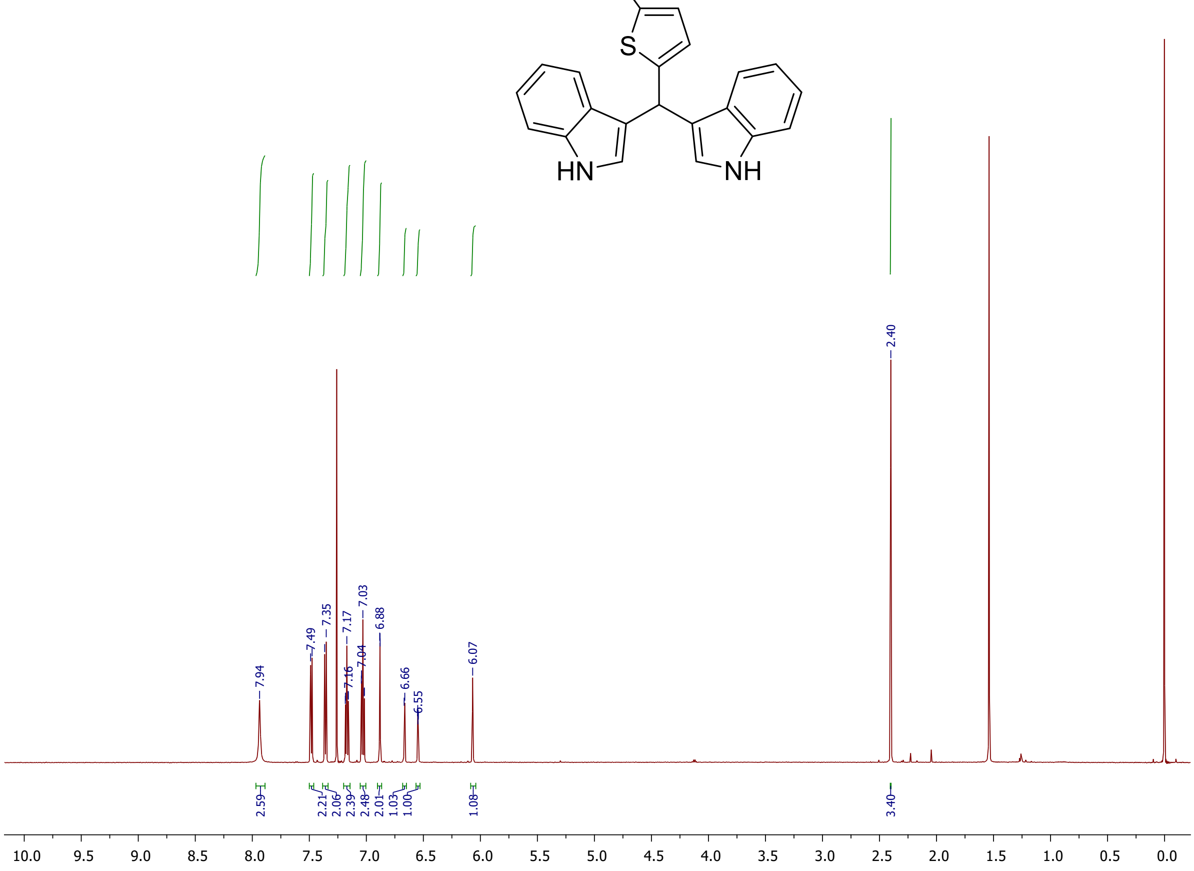

^{13}C NMR ($CDCl_3$, 150 MHz) of compound **12**.

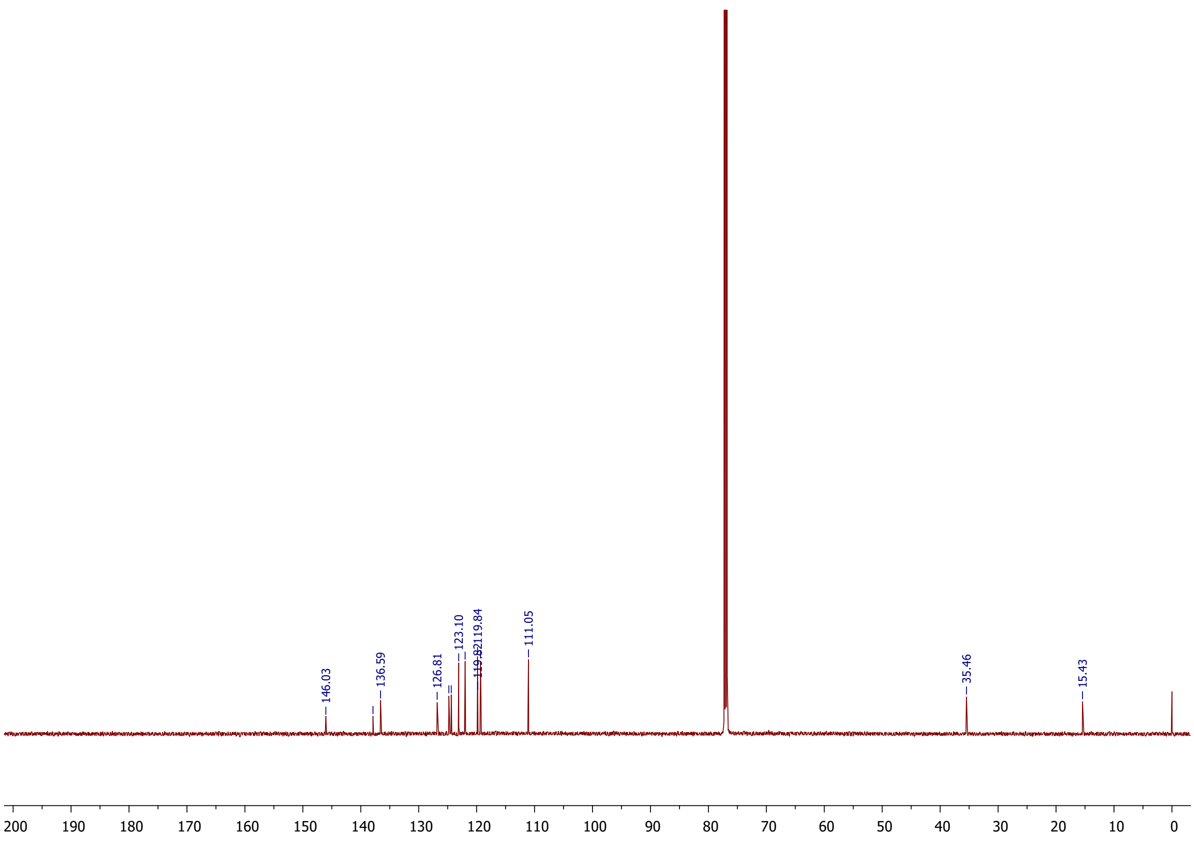

^{1}H NMR ($CDCl_3$, 600 MHz) of compound **13**.

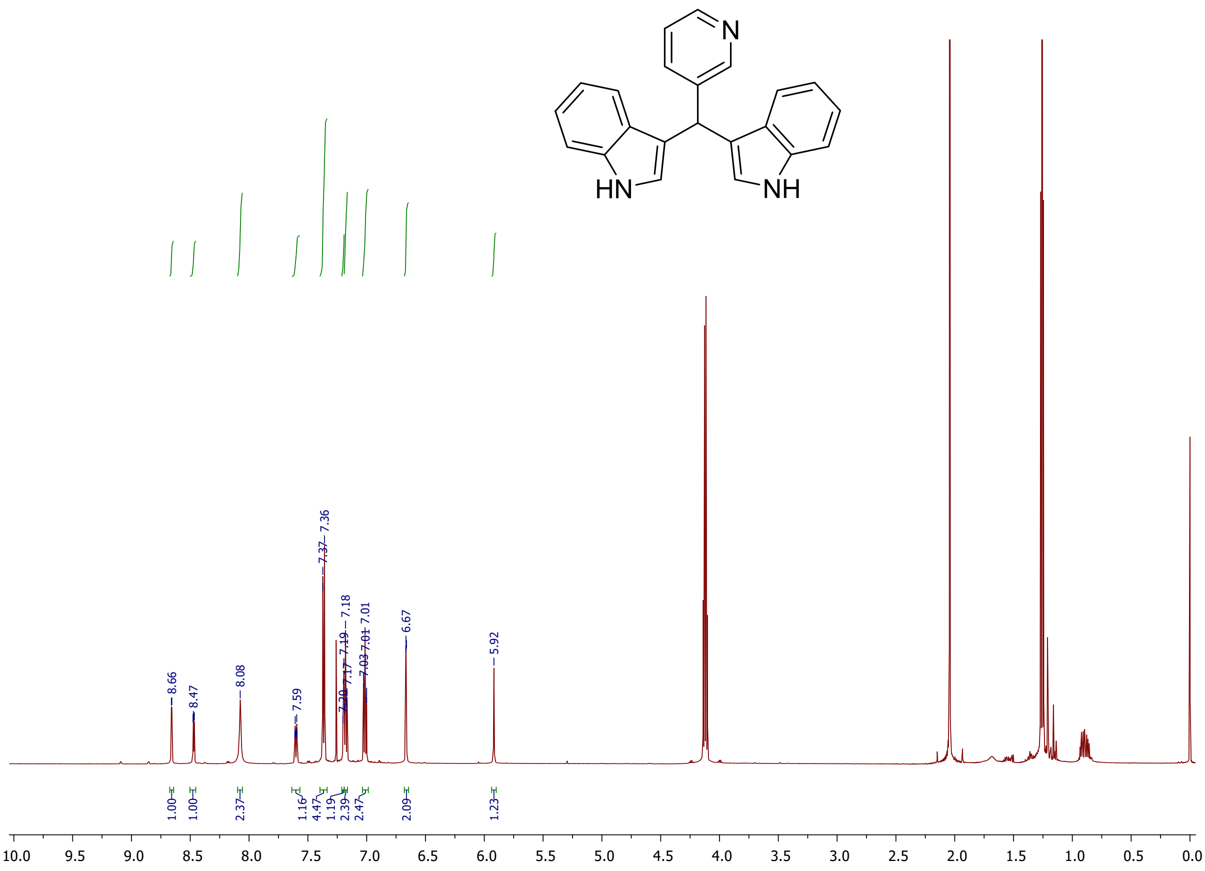

^{13}C NMR ($CDCl_3$, 150 MHz) of compound **13**.

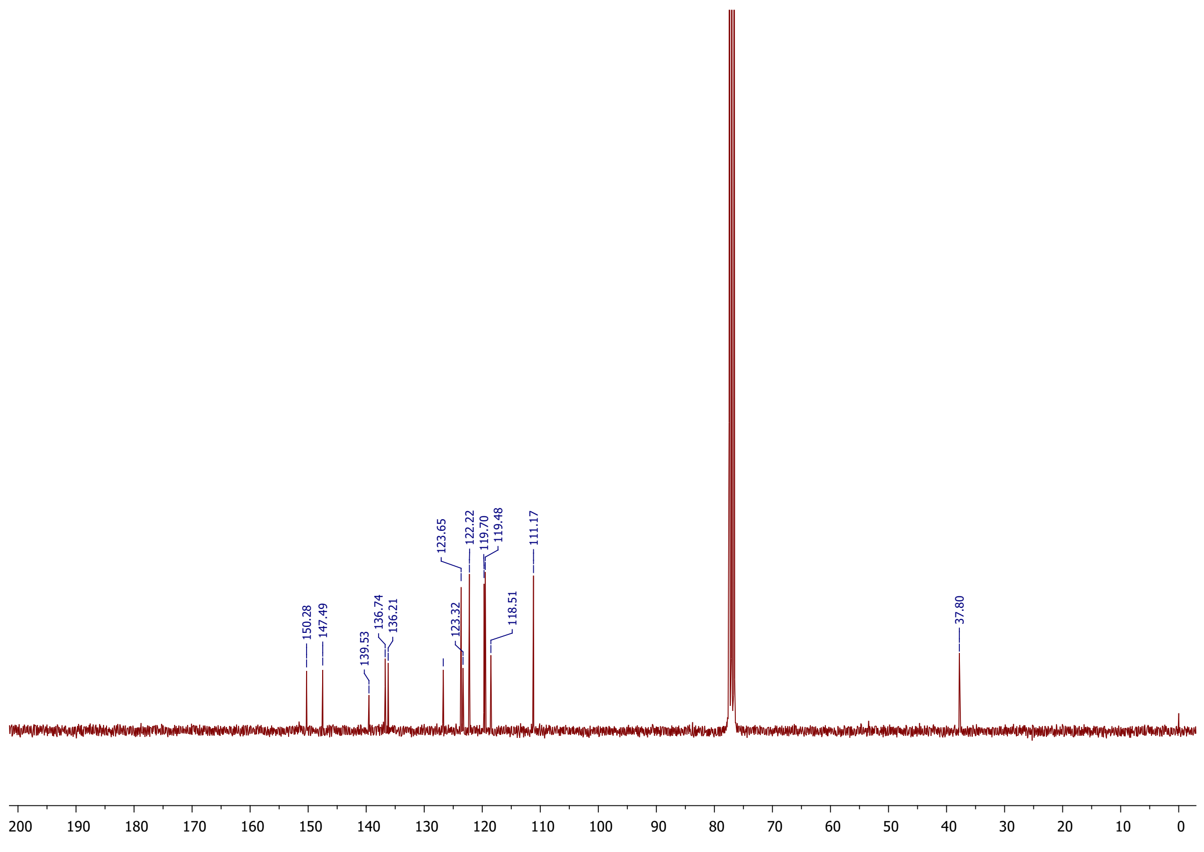

Cartesian coordinates of optimized ligands docked into DNA gyrase B and 14α-sterol demethylase

Molecule 3

C	0.049446	-0.374003	0.010439
C	-0.006034	0.026199	1.348251
C	1.144579	0.448374	2.017930
C	2.380656	0.482334	1.365380
C	2.440869	0.080345	0.037839
C	1.278017	-0.343937	-0.640430
C	1.661522	-0.700973	-2.012824
C	2.986319	-0.495078	-2.177532
C	3.567094	0.006813	-0.919880
C	4.850304	0.314784	-0.639605
H	3.270266	0.817069	1.892206
H	1.078327	0.755610	3.056907
H	-0.955661	0.010305	1.874240
H	-0.847789	-0.699310	-0.508067
H	5.079306	0.589878	0.392075
C	5.969466	0.346153	-1.588108
H	0.974281	-1.085123	-2.757724
H	3.557907	-0.698680	-3.073666
C	7.249751	-0.069183	-1.171989
C	8.341599	0.015728	-2.036481
C	8.171406	0.509870	-3.323494
C	6.911704	0.926361	-3.758317
C	5.828984	0.847355	-2.892638
O	7.486663	-0.568626	0.067344
H	9.310806	-0.319321	-1.681749
H	9.026742	0.572040	-3.988971
H	6.780050	1.326562	-4.757798
H	4.855978	1.213112	-3.205531
H	6.653743	-0.771207	0.517879

Molecule 5

C	-0.009565	-0.233097	0.013521
C	0.003485	-0.029015	1.395923
C	1.202834	0.196607	2.074990
C	2.420290	0.224892	1.388063
C	2.411977	0.017027	0.015365
C	1.200271	-0.208023	-0.671770
C	1.517332	-0.391844	-2.093946
C	2.849986	-0.270426	-2.278241
C	3.504390	-0.003285	-0.984654
C	4.812133	0.155527	-0.694444
H	3.348142	0.405331	1.924104
H	1.189903	0.352880	3.149092
H	-0.930219	-0.044727	1.949670
H	-0.944092	-0.406033	-0.512242
H	5.088316	0.236875	0.356637
C	5.934171	0.201714	-1.635622

H	0.782143	-0.609898	-2.859913
H	3.372323	-0.396678	-3.217176
C	7.190141	-0.257399	-1.209720
N	8.282160	-0.273255	-1.972263
C	8.169653	0.196715	-3.220067
C	6.983067	0.707451	-3.739336
C	5.851151	0.717907	-2.935098
H	9.071489	0.178243	-3.827038
H	6.955113	1.102927	-4.748521
H	4.924384	1.150949	-3.296736
H	7.300108	-0.636433	-0.194751

Molecule 6

C	-2.23680	0.92280	-0.11150
C	-3.62350	0.94030	-0.06620
C	-4.30650	-0.27100	0.08290
C	-3.60820	-1.47640	0.18370
C	-2.21300	-1.49890	0.13880
C	-1.53430	-0.29090	-0.00800
H	-4.17200	1.87510	-0.14510
H	-5.39160	-0.27500	0.12000
H	-4.15760	-2.40590	0.29800
H	-1.67280	-2.43860	0.21720
C	-0.08970	-0.00440	-0.08450
C	0.07930	1.32310	-0.22840
C	-1.24270	2.04550	-0.26260
H	-1.38450	2.59540	-1.20170
H	-1.32930	2.78390	0.54440
H	1.03440	1.83050	-0.31060
C	0.94980	-1.09020	-0.01180
C	2.36200	-0.59710	-0.03370
C	3.36120	-0.74700	-0.97210
N	2.87580	0.15780	0.98820
C	4.51230	-0.05770	-0.49090
C	4.17650	0.49210	0.72520
H	0.76710	-1.68540	0.89380
H	0.80880	-1.77850	-0.85350
H	3.26690	-1.29600	-1.89940
H	5.47370	0.02340	-0.97850
H	4.74910	1.08670	1.42150
H	2.35830	0.43730	1.81090

Molecule 8

C	0.255844	-0.607946	-0.171672
C	0.149428	-0.721658	1.207086
C	1.134463	-0.135004	2.008235
C	2.205524	0.552815	1.433336
C	2.316891	0.669402	0.046543
C	1.335197	0.083534	-0.749947
H	-0.681999	-1.255039	1.659980
H	1.066061	-0.214583	3.088895
H	2.959432	1.001579	2.073119

H	3.151789	1.204593	-0.397872
C	1.188370	0.041376	-2.216810
C	0.069583	-0.642954	-2.517292
C	2.178239	0.693747	-3.143182
C	-0.633544	-1.123531	-1.273996
H	-0.297249	-0.838263	-3.519198
H	-1.653676	-0.726133	-1.198438
C	1.860748	0.501081	-4.603010
C	2.179728	-0.709843	-5.237567
C	1.871910	-0.912928	-6.582945
C	1.234369	0.091174	-7.308403
C	0.904955	1.296848	-6.695798
C	1.220449	1.487768	-5.350440
H	2.131937	-1.857288	-7.054826
H	0.998490	-0.075479	-8.354904
H	0.409284	2.081913	-7.257047
H	0.969483	2.426096	-4.861819
H	2.217544	1.766401	-2.916683
H	3.175244	0.293613	-2.920257
O	2.798583	-1.658516	-4.484974
H	2.989706	-2.431345	-5.037181
H	-0.724913	-2.216886	-1.250365

Molecule 11

C	0.118852	0.015009	0.064208
H	0.281816	0.073657	1.151031
C	1.424900	-0.480291	-0.570676
C	-0.992188	-0.982587	-0.159987
C	-0.252550	1.392377	-0.417324
C	0.346571	2.127955	-1.406985
N	-0.280723	3.345783	-1.540157
C	-1.306279	3.427745	-0.633141
C	-1.323284	2.211546	0.094729
H	-0.019035	4.065910	-2.198338
H	1.189821	1.883250	-2.037750
H	1.314335	-0.555790	-1.657344
H	2.252124	0.201968	-0.351842
H	1.684356	-1.471298	-0.188768
C	-2.038441	-0.900284	-1.041399
N	-2.803738	-2.042667	-0.977299
C	-2.268961	-2.893992	-0.044674
C	-1.119639	-2.260872	0.493571
H	-3.639260	-2.214527	-1.518176
H	-2.307108	-0.100730	-1.718003
C	-0.377586	-2.924720	1.486700
C	-2.691148	-4.160321	0.379018
C	-1.938638	-4.788675	1.357892
C	-0.792107	-4.177345	1.908675
H	-3.574678	-4.626717	-0.045730
H	-2.237176	-5.771511	1.709401
H	-0.229461	-4.701053	2.675269
H	0.505865	-2.459441	1.916435
C	-2.290027	2.031299	1.099611

C	-2.217875	4.461086	-0.382947
C	-3.155623	4.255061	0.615744
C	-3.192411	3.051622	1.351905
H	-2.326204	1.104625	1.666302
H	-3.943498	2.927399	2.125851
H	-3.877624	5.035170	0.836778
H	-2.186782	5.385254	-0.951720

Molecule 13

C	-0.627564	0.064608	0.104098
H	-0.871189	0.155875	1.171835
C	0.888506	-0.084881	0.028109
C	-1.340159	-1.155356	-0.427025
C	-1.045231	1.360130	-0.550958
C	-1.133814	1.641402	-1.891539
N	-1.463234	2.962774	-2.073307
C	-1.591319	3.572629	-0.851918
C	-1.326920	2.592542	0.138113
H	-1.607078	3.407754	-2.968989
H	-0.980856	0.996882	-2.746323
C	-2.367279	-1.222540	-1.332929
N	-2.760522	-2.527939	-1.506674
C	-1.995137	-3.342877	-0.712559
C	-1.085031	-2.513586	-0.010220
H	-3.494429	-2.834431	-2.129407
H	-2.866778	-0.430258	-1.870950
C	-0.182354	-3.106704	0.891213
C	-2.025261	-4.732751	-0.545477
C	-1.123248	-5.286178	0.348005
C	-0.210789	-4.480975	1.061892
H	-2.731229	-5.346872	-1.095797
H	-1.118601	-6.360602	0.503295
H	0.480089	-4.950804	1.754964
H	0.525341	-2.495844	1.445025
C	-1.380624	2.963199	1.493978
C	-1.914661	4.896216	-0.527887
C	-1.963009	5.228588	0.815457
C	-1.696966	4.271486	1.818486
H	-1.177565	2.233623	2.273552
H	-1.742498	4.570929	2.860901
H	-2.208790	6.245919	1.103766
H	-2.116949	5.628352	-1.303375
C	1.530785	-0.608310	-1.094210
C	2.918408	-0.676996	-1.110466
C	3.622297	-0.219157	0.001410
N	3.027924	0.282773	1.087336
C	1.692376	0.341257	1.085927
H	0.946184	-0.962998	-1.939802
H	3.451148	-1.080718	-1.964677
H	4.708851	-0.261993	0.019275

Estimated free energies of binding obtained by molecular docking

Table S1. Free energies of binding, ΔG_{bind} obtained by molecular docking of listed molecules against DNA gyrase B, along with the number of conformational clusters and number of conformations within the most populated cluster

Compound	ΔG_{bind}/kcal mol^{-1} lowest	highest	Number of distinctive conformational clusters	Number of conformations within the most populated cluster
**3**	-7.31	-5.18	7	80
**5**	-6.97	-5.13	4	97
**8**	-6.93	-5.03	11	36
**11**	-7.26	-6.08	10	34
**13**	-7.88	-5.02	29	23
**Amoxicillin**	-6.22	-2.99	68	5

*number of docking runs = 100

Table S2. Free energies of binding, ΔG_{bind} obtained by molecular docking of listed molecules against 14α-sterol demethylase, along with the number of conformational clusters and number of conformations within the most populated cluster

Compound	ΔG_{bind}/kcal mol^{-1} lowest	highest	Number of distinctive conformational clusters	Number of conformations within the most populated cluster
**3**	-8.20	-6.80	7	33
**5**	-8.38	-7.75	5	58
**6**	-7.62	-7.07	4	69
**Ketoconazole**	-9.45	-4.49	71	8

*number of docking runs = 100
